# HIV-Positive Patients on Antiretroviral Therapy Have an Altered Mucosal Intestinal but Not Oral Microbiome

**DOI:** 10.1128/spectrum.02472-22

**Published:** 2022-12-13

**Authors:** Jingjing Meng, Junyi Tao, Yaa Abu, Daniel Andrew Sussman, Mohit Girotra, Dido Franceschi, Sabita Roy

**Affiliations:** a Department of Surgery, Sylvester Comprehensive Cancer Center, University of Miami, Miami, Florida, USA; b Department of Gastroenterology, University of Miami Medical Group, Miami, Florida, USA; U. S. Food and Drug Administration, National Center for Toxicological Research

**Keywords:** 16S RNA, HIV, antiretroviral therapy, human microbiome, intestinal microbiome, oral microbiome

## Abstract

This study characterized compositional and functional shifts in the intestinal and oral microbiome in HIV-positive patients on antiretroviral therapy compared to HIV-negative individuals. Seventy-nine specimens were collected from 5 HIV-positive and 12 control subjects from five locations (colon brush, colon wash, terminal ileum [TI] brush, TI wash, and saliva) during colonoscopy and at patient visits. Microbiome composition was characterized using 16S rRNA sequencing, and microbiome function was predicted using bioinformatics tools (PICRUSt and BugBase). Our analysis indicated that the β-diversity of all intestinal samples (colon brush, colon wash, TI brush, and TI wash) from patients with HIV was significantly different from patients without HIV. Specifically, bacteria from genera *Prevotella*, *Fusobacterium*, and *Megasphaera* were more abundant in samples from HIV-positive patients. On the other hand, bacteria from genera *Ruminococcus, Blautia*, and *Clostridium* were more abundant in samples from HIV-negative patients. Additionally, HIV-positive patients had higher abundances of biofilm-forming and pathogenic bacteria. Furthermore, pathways related to translation and nucleotide metabolism were elevated in HIV-positive patients, whereas pathways related to lipid and carbohydrate metabolism were positively correlated with samples from HIV-negative patients. Our analyses further showed variations in microbiome composition in HIV-positive and negative patients by sampling site. Samples from colon wash, colon brush, and TI wash were significant between groups, while samples from TI brush and saliva were not significant. Taken together, here, we report altered intestinal microbiome composition and predicted function in patients with HIV compared to uninfected patients, though we found no changes in the oral microbiome.

**IMPORTANCE** Over 37 million people worldwide are living with HIV. Although the availability of antiretroviral therapy has significantly reduced the number of AIDS-related deaths, individuals living with HIV are at increased risk for opportunistic infections. We now know that HIV interacts with the trillions of bacteria, fungi, and viruses in the human body termed the microbiome. Only a limited number of previous studies have compared variations in the oral and gastrointestinal microbiome with HIV infection. Here, we detail how the oral and gastrointestinal microbiome changes with HIV infection, having used 5 different sampling sites to gain a more comprehensive view of these changes by location. Our results show site-specific changes in the intestinal microbiome associated with HIV infection. Additionally, we show that while there were significant changes in the intestinal microbiome, there were no significant changes in the oral microbiome.

## INTRODUCTION

Human immunodeficiency virus (HIV) infection is characterized by profound depletion of circulating and tissue-resident CD4-positive (CD4^+^) T cells in gut-associated lymphoid tissue and a chronic inflammatory state (1). Although the overall survival of HIV patients has significantly improved since the introduction of antiretroviral therapy (ART), HIV-infected adults still have an increased risk of cardiovascular, liver, kidney, bone, and neurologic diseases (2), which are partially driven by microbial translocation and subsequent immune activation (3, 4).

In recent years, multiple groups have characterized the microbiome in the oral cavity or intestines in patients with HIV infection, though relatively few have examined both areas in the same patient. Although the oral cavity and the intestines are part of the gastrointestinal tract, they harbor distinct microbial communities, with the oral cavity dominated by *Firmicutes*, while the stool microbiota is mostly abundant in *Bacteroidetes* (5, 6). These unique communities have been attributed to gastric acid in the stomach and bile acids in the duodenum (5, 7, 8). Thus, while HIV is known to cause profound changes to the gastrointestinal system at large (9–11), recognizing site-specific differences is key to fully appreciating the microbial landscape altered with HIV infection. Still, the majority of previous studies investigating intestinal microbial alterations with HIV infection have only utilized one sampling site, with stool/stool swab (12, 13) samples being the most common, followed by rectal sponges (14) and rectosigmoid biopsy specimen (15). Similarly, in the oral cavity, the most common sampling sites utilized are saliva (16–19) and oral washes (20–22), with some studies using plaque samples (23, 24) or biofilm (25), but relatively few using multiple areas. Despite our understanding of site-specific microbial communities along the gastrointestinal (GI) tract, to date, only one study has investigated the intestinal microbiome at different sites, including the terminal ileum (TI), right colon, left colon, and feces (26); however, the oral microbiome was not evaluated in that study.

Characterization of the intestinal microbiome, in particular, sheds light on the importance of sampling methods in identifying distinct microbial communities in many disease states. Gastrointestinal tract commensal bacteria consist of contents within the transient luminal compartment and the mucosal adherent compartment (27). While most studies investigating the intestinal microbiota in humans have often used fecal samples because they are easily collected, the fecal microbiota is substantially variable between individuals and is often influenced by food/ingested materials, which limits our ability to identify specific disease-associated microbes (27, 28). On the other hand, the mucosa-associated microbiota is the more stable adherent compartment that adheres to the mucosal surface of the GI tract, though the main means of characterizing this compartment are through colonoscopic biopsies, which are relatively invasive and limit their use (27, 28). Notably, clinical studies of microbiome changes in HIV infection have shown differential bacterial microbiome phenotypes in intestinal biopsy specimens, particularly in the terminal ileum, compared to fecal samples from the same individuals (26, 29). These mucosal biopsy specimens also have permitted the examination of microbes that are most closely associated with the immune system (30). Additionally, during colonoscopic biopsy specimens, flushing the mucosal surface with sterile water allows for the mucosal-luminal interface to be sampled by washing off and collecting the loose mucus layer on the surface of the intestinal wall (28). However, few studies have compared mucosal-luminal interface sampling to biopsy specimens, stool, or saliva.

In this study, we aimed to characterize compositional and functional shifts in the mucosal intestinal microbiome and the oral microbiome in HIV-positive individuals on ART compared to uninfected individuals by using 16S rRNA sequencing. Here, we used multiple sampling sites, including brush samples and washes (colon brush, colon wash, terminal ileum brush, and terminal ileum wash) during colonoscopic procedures, as well as saliva samples at patient visits, to gain a comprehensive view of intestinal and oral microbiome changes in the context of HIV infection.

## RESULTS

### Patient selection.

A total of 17 patients were enrolled in this study. Five patients were diagnosed with HIV, and 12 patients were HIV negative. All HIV patients were on antiretroviral therapy. We refer to them as HIV-positive patients throughout the text for brevity. Patient characteristics are summarized in Table 1, and detailed clinical characteristics for each subject can be found in Table S1 in the supplemental material. Notably, there was no significant difference (*P* = 0.5) in the mean age of patients between groups, though the female/male ratio significantly differed (*P* = 0.01). Ethnicity in both groups was not significantly different (*P* = 0.60), but Hispanics were overrepresented. Additionally, the incidence of obesity in both groups was not significantly different (*P* = 0.53). Similarly, the incidence of diabetes was not significantly different between groups (*P* = 0.83).

**TABLE 1:** Patient characteristics

Characteristic	Data for:	
	HIV-negative patients	HIV-positive patients on ART
No. of subjects	12	5
Mean age (yrs)	55	51
No. female/no. male	8/4	0/5
Ethnicity (no.)		
Hispanic	8	4
Non-Hispanic	4	1

Samples collected in this study were obtained from terminal ileum (TI) wash, TI brush, colon wash, and colon brush of patients undergoing colonoscopy for a comprehensive examination of mucosa-associated microbiota (brush samples), as well as the mucosal-luminal interface (wash samples). Saliva samples were obtained directly from patients spitting into sterile containers to concomitantly survey the oral microbiome from the same patient.

### Altered intestinal microbiome diversity and composition in patients with HIV on ART.

**FIG 1:**
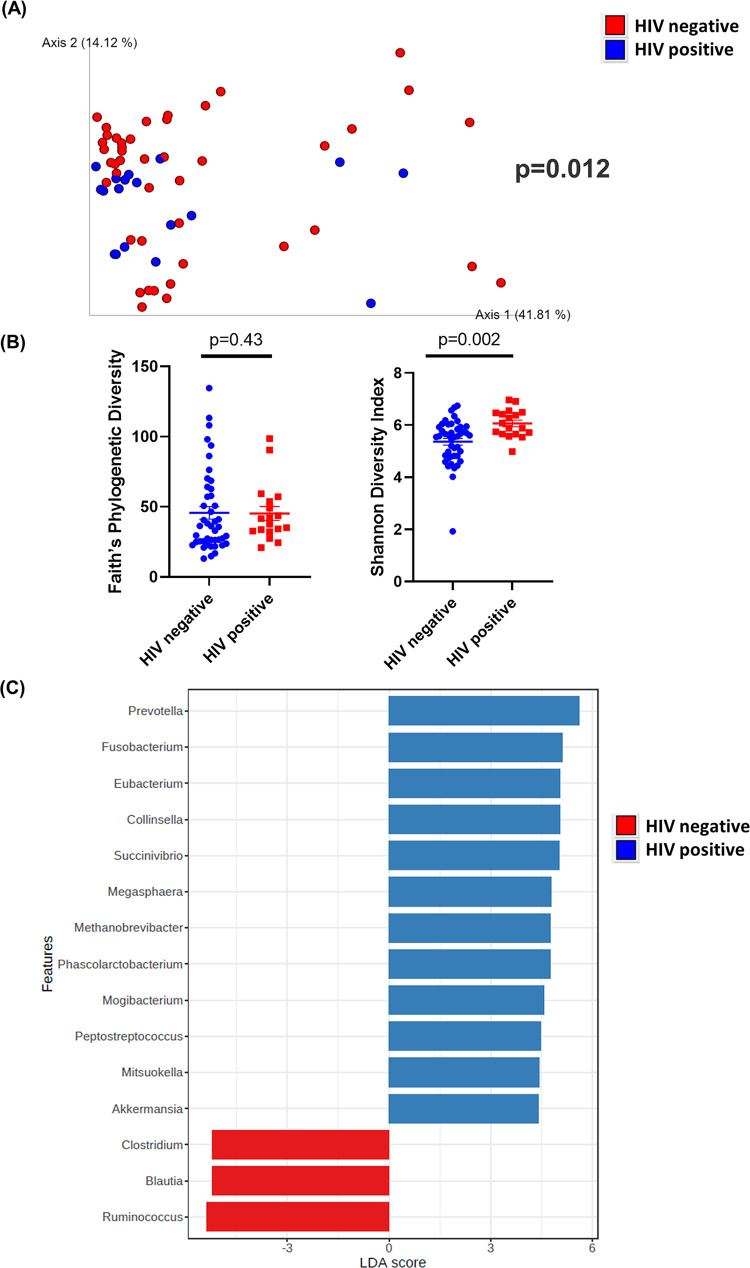
Diversity and composition analysis of the gut samples. Samples were grouped by HIV-negative (*n* = 44) and -positive (*n* = 18) status. (A) Principal-coordinate analysis (PCoA) plot of weighted UniFrac distances (metrics of β-diversity). *P* = 0.012. (B) Faith’s phylogenic diversity and Shannon diversity index (metrics of α-diversity) at a sequencing depth of 80,000 reads. Samples were grouped by HIV-negative (*n* = 44) and -positive (*n* = 18) status. Error bars represent SEM. (C) Linear discriminant analysis effect size (LEfSe) analysis of top discriminative bacteria genera between gut samples from HIV-positive and -negative patients.

On average, we obtained 178,608 sequence reads per intestinal sample and 176,926 sequence reads per saliva sample (Table S2). Among all intestinal samples, 4,310 unique amplicon sequence variants (ASVs) were identified (Table S3). Overall, our results showed that HIV significantly alters the diversity of the intestinal microbiome. β-Diversity was assessed by weighted UniFrac distances and visualized with principal-coordinate analysis (PCoA) plots. Analysis of β-diversity showed that all intestinal samples (colon brush, colon wash, TI brush, and TI wash) from patients with HIV significantly clustered apart from intestinal samples from patients without HIV (*P* = 0.012) (Fig. 1A). Additionally, the α-diversity was measured by Faith’s phylogenetic diversity and Shannon diversity index. At a sequencing depth of 80,000 reads, Faith’s phylogenetic diversity was not significantly different between patients with or without HIV (*P* = 0.43) (Fig. 1B). However, when α-diversity was measured by Shannon diversity index, intestinal samples from patients with HIV exhibited higher (*P* = 0.002) richness and evenness than those from patients without HIV (Fig. 1B).

The bar plots of bacterial composition at phylum and genus levels in the gut samples are shown in Fig. S1. Linear discriminant analysis (LDA) effect size (LEfSe) analysis was performed among intestinal samples to determine the bacterial taxa that were differentially enriched. Bacteria from the genera *Prevotella*, *Fusobacterium*, *Eubacterium*, *Collinsella*, *Megasphaera*, *Mogibacterium*, and *Mitsuokella* were more abundant in samples from HIV-positive patients. On the other hand, bacteria from the genera *Ruminococcus*, *Blautia*, and *Clostridium* were more abundant in samples from HIV-negative patients (Fig. 1C).

### Altered predicted intestinal microbiome function in patients with HIV on ART.

**FIG 2:**
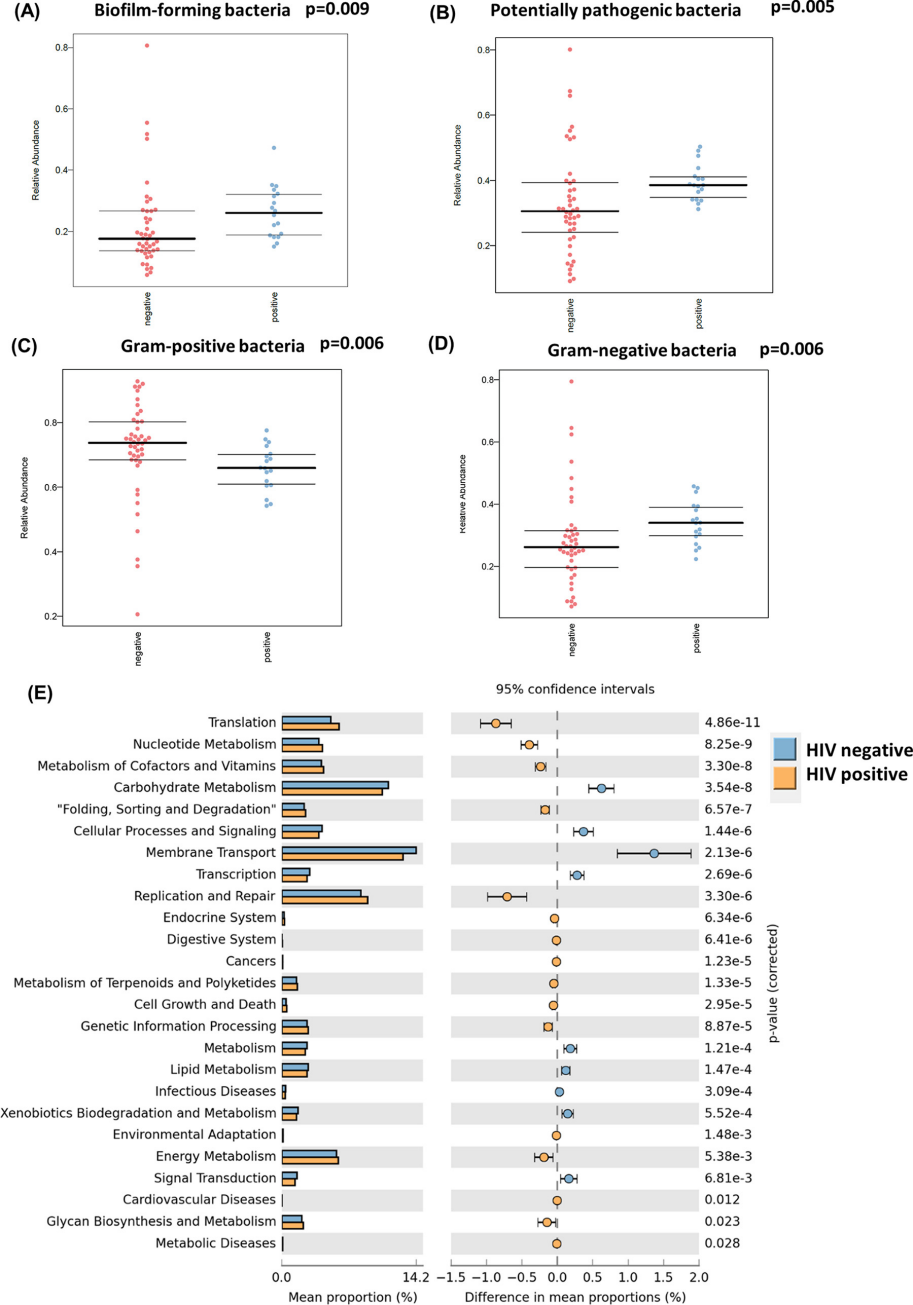
Predictive functional analysis of the gut samples. (A) BugBase predicted the relative abundance of biofilm-forming bacteria. Samples grouped by HIV-negative (*n* = 44) and -positive status (*n* = 18); *P* = 0.009. (B) BugBase predicted the relative abundance of potentially pathogenic bacteria. Samples grouped by HIV-negative (*n* = 44) and -positive (*n* = 18) status; *P* = 0.005. (C) BugBase predicted the relative abundance of Gram-positive bacteria. Samples grouped by HIV-negative (*n* = 44) and -positive (*n* = 18) status; *P* = 0.006. (D) BugBase predicted the relative abundance of Gram-negative bacteria. Samples grouped by HIV-negative (*n* = 44) and -positive (*n* = 18) status; *P* = 0.006. (E) The KEGG pathway of gut microbiota was predicted using Phylogenetic Investigation of Communities by Reconstruction of Unobserved States (PICRUSt). Data are presented in a bar plot with 95% confidence intervals and *P* values between gut samples from HIV-positive and -negative patients.

The BugBase algorithm was used to predict high-level phenotypes present in intestinal microbiome samples by using 16S amplicon data. The BugBase phenotype predicted the abundance of Gram-positive, Gram-negative, biofilm-forming, and potentially pathogenic bacteria. Intestinal samples from HIV-positive patients had a higher abundance of both biofilm-forming (*P* = 0.009) and pathogenic bacteria (*P* = 0.005) (Fig. 2A and B). Additionally, HIV-positive samples had higher percentages of Gram-negative bacteria (*P* = 0.006) and lower percentages of Gram-positive bacteria (*P* = 0.006) (Fig. 2C and D).

The microbial metagenome was predicted with the Phylogenetic Investigation of Communities by Reconstruction of Unobserved States (PICRUSt) algorithm, and functions were categorized with KEGG pathways to further elucidate the specific changes in microbial pathways. STAMP was used for identifying pathways that were differentially abundant between HIV-positive and negative patients. In total, 41 KEGG level 2 pathways were predicted among all intestinal samples (Table S4). Pathways related to translation, nucleotide metabolism, cofactors and vitamin metabolism, and replication and repair were positively correlated with samples from HIV-positive patients (Fig. 2E). On the other hand, pathways related to lipid and carbohydrate metabolism, membrane transport, signaling transduction, and cellular processes and signaling were positively correlated with samples from HIV-negative patients (Fig. 2E).

### Differences in HIV-associated intestinal microbiome diversity, composition, and predicted function by sampling site.

**FIG 3:**
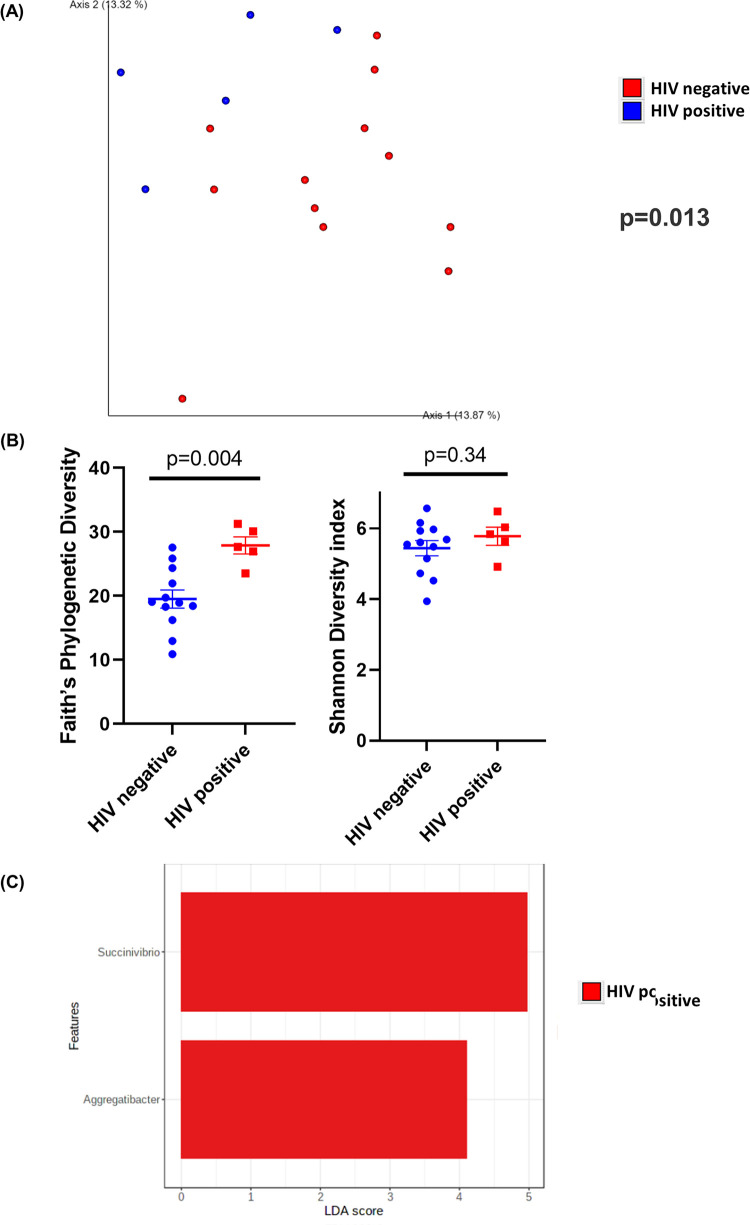
Diversity and composition analysis of the colon wash samples. (A) Principal-coordinate analysis (PCoA) plot of unweighted UniFrac distances (metrics of β-diversity). Samples grouped by HIV-negative (*n* = 12) and -positive (*n* = 5) status; *P* = 0.013. (B) Faith’s phylogenetic diversity and Shannon diversity index (metrics of α-diversity) at a sequencing depth of 80,000 reads. Samples were grouped by HIV-negative (*n* = 12) and -positive (*n* = 5) status. Error bars represent SEM. (C) Linear discriminant analysis effect size (LEfSe) analysis of top discriminative bacteria genera between gut samples from HIV-positive and -negative patients.

Intestinal samples were collected from 4 different sampling sites. Following this, we then compared samples from HIV-positive and HIV-negative patients at each sampling site (colon wash, colon brush, TI wash, and TI brush). In colon wash samples, the β-diversity, measured using unweighted UniFrac distances, between HIV-positive and HIV-negative samples was significantly different (*P* = 0.013) (Fig. 3A). Additionally, in colon wash, HIV-positive samples had higher richness (*P* = 0.004) than HIV-negative samples when the α-diversity was measured by Faith’s phylogenetic diversity (Fig. 3B). However, there was no difference (*P* = 0.34) in sample richness or evenness between HIV-positive and -negative colon wash samples when α-diversity was measured by Shannon diversity index (Fig. 3B). Furthermore, LEfSe analysis showed that bacteria from the genera *Succinivibrio* and *Aggregatibacter* were more abundant in HIV-positive colon wash samples (Fig. 3C). No bacteria were enriched in samples from HIV-negative patients. Colon wash samples from HIV-positive patients had similar abundance of both biofilm-forming (*P* = 0.16) and pathogenic bacteria (*P* = 0.06) (Fig. S2A and B). Additionally, HIV-positive samples had higher percentages of Gram-negative bacteria (*P* = 0.02) and lower percentages of Gram-positive bacteria (*P* = 0.02) (Fig. S2C and D). Pathways related to translation, nucleotide metabolism, and genetic information processing were positively correlated with samples from HIV-positive patients (Fig. S2E). Pathways related to carbohydrate metabolism and transcription were positively correlated with samples from HIV-negative patients (Fig. S2E).

**FIG 4:**
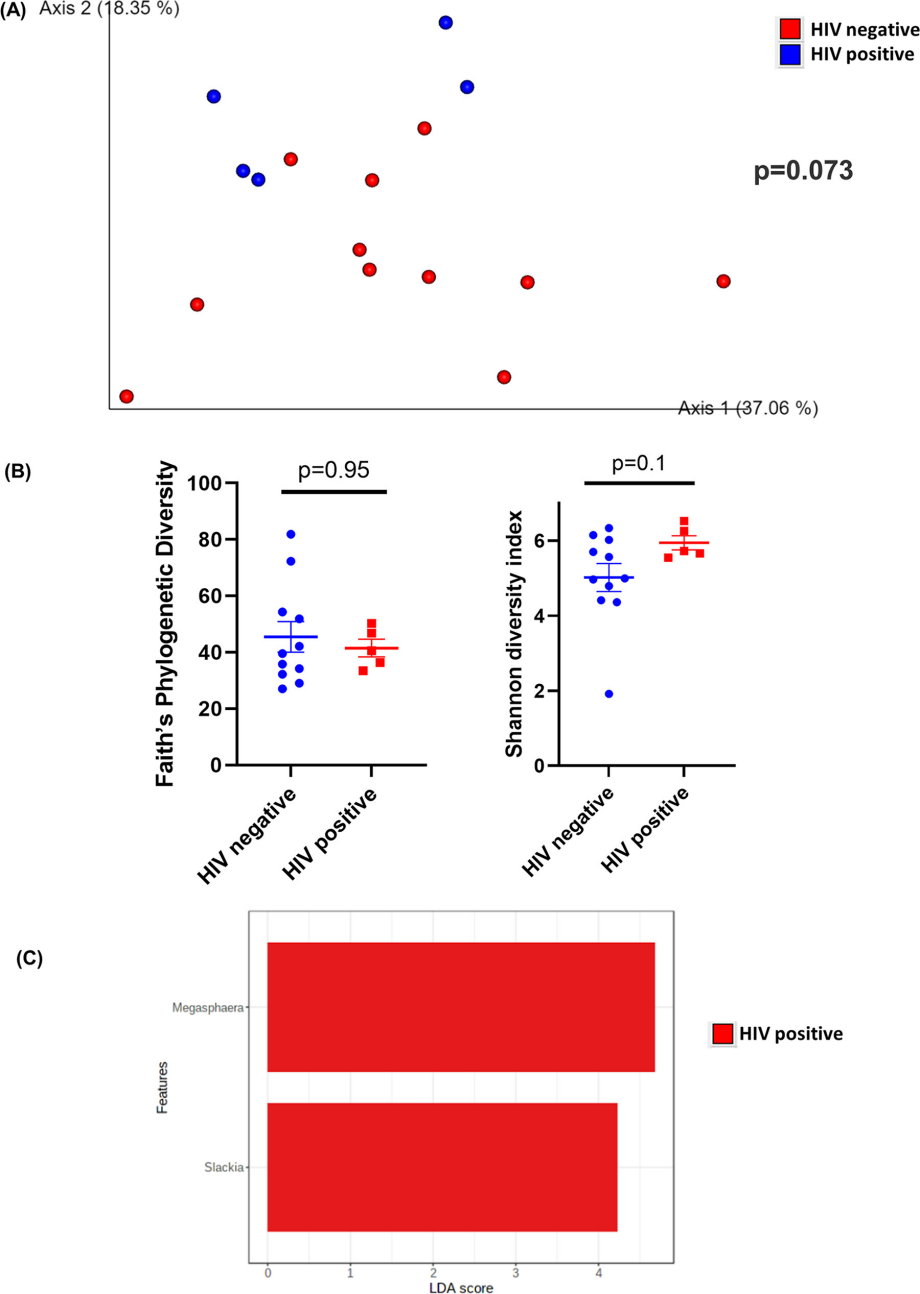
Diversity and composition analysis of the colon brush samples. (A) Principal-coordinate analysis (PCoA) plot of weighted UniFrac distances (metrics of β-diversity). Samples grouped by HIV-negative (*n* = 12) and -positive (*n* = 5) status, *P* = 0.073. (B) Faith’s phylogenetic diversity and Shannon diversity index (metrics of α-diversity) at a sequencing depth of 80,000 reads. Samples were grouped by HIV-negative (*n* = 12) and -positive (*n* = 5) status. Error bars represent SEM. (C) Linear discriminant analysis effect size (LEfSe) analysis of top discriminative bacteria genera between gut samples from HIV-positive and -negative patients.

In colon brush samples, HIV-positive and HIV-negative samples showed a tendency to cluster differently (*P* = 0.073) when β-diversity was measured using weighted UniFrac distances (Fig. 4A). Additionally, there was no difference in Faith’s phylogenetic diversity (*P* = 0.95) or Shannon diversity index (*P* = 0.1) between colon brush samples (Fig. 4B). Notably, bacteria from *Megasphaera* and *Slackia* were enriched in HIV-positive colon brush samples (Fig. 4C). No bacteria were enriched in samples from HIV-negative patients. Colon brush samples from HIV-positive patients had a similar abundance of biofilm-forming bacteria (*P* = 0.11) (Fig. S3A). However, colon brush samples from HIV-negative patients had a higher abundance of potentially pathogenic bacteria (*P* = 0.008) (Fig. S3B). There was no difference in the abundance of Gram-positive and Gram-negative bacteria (*P* = 0.44) (Fig. S3C and D). Pathways related to translation, nucleotide metabolism, cofactors and vitamin metabolism, and replication and repair were positively correlated with samples from HIV-positive patients (Fig. S3E). Pathways related to membrane transport, cellular processes and signaling, lipid and carbohydrate metabolism, and transcription were positively correlated with samples from HIV-negative patients (Fig. S3E).

**FIG 5:**
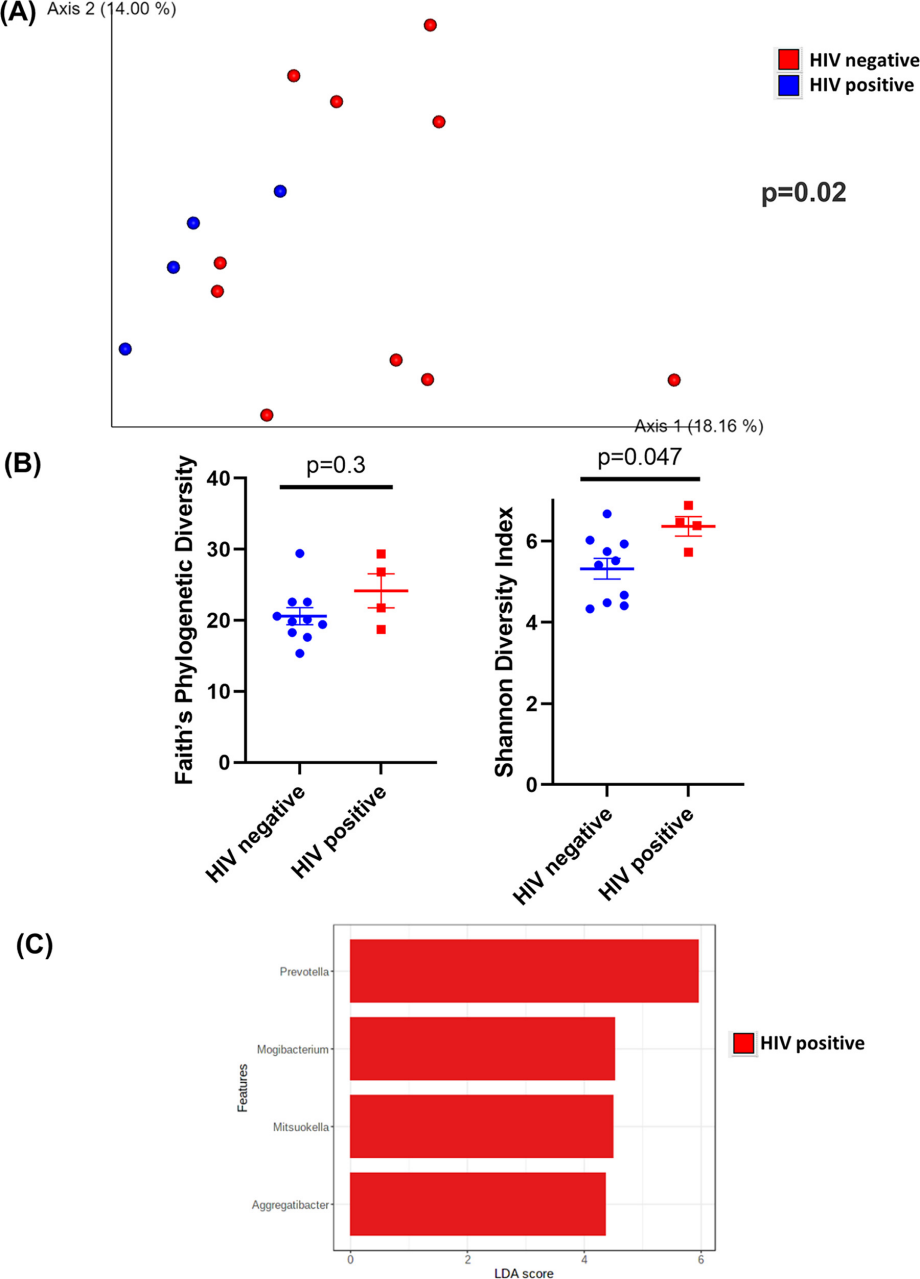
Diversity and composition analysis of the TI wash samples. (A) Principal-coordinate analysis (PCoA) plot of unweighted UniFrac distances (metrics of β-diversity). Samples grouped by HIV-negative (*n* = 10) and -positive (*n* = 4) status; *P* = 0.02. (B) Faith’s phylogenetic diversity and Shannon diversity index (metrics of α-diversity) at a sequencing depth of 80,000 reads. Samples were grouped by HIV-negative (*n* = 10) and -positive (*n* = 4) status. Error bars represent SEM. (C) Linear discriminant analysis effect size (LEfSe) analysis of top discriminative bacteria genera between intestinal samples from HIV-positive and -negative patients.

In TI wash samples, HIV-positive and HIV-negative samples were significantly different (*P* = 0.02) as assessed by unweighted UniFrac distances (Fig. 5A). There was no difference (*P* = 0.3) in sample richness or evenness when the α-diversity was measured by Faith’s phylogenetic diversity (Fig. 5B). However, HIV-positive TI wash samples had higher (*P* = 0.047) α-diversity when α-diversity was measured by the Shannon diversity index (Fig. 5B). Notably, bacteria from *Prevotella*, *Mogibacterium*, *Mitsuokella*, and *Aggregatibacter* were enriched in HIV-positive TI wash samples (Fig. 5C). No bacteria were enriched in samples from HIV-negative patients. TI wash samples from HIV-positive patients had a similar abundance of biofilm-forming bacteria (*P* = 0.14) (Fig. S4A). However, TI wash samples from HIV-positive patients had a higher abundance of potentially pathogenic bacteria (*P* = 0.04) (Fig. S4B). HIV-positive samples had higher percentages of Gram-negative bacteria (*P* = 0.04) and lower percentages of Gram-positive bacteria (*P* = 0.04) (Fig. S4C and D). Pathways related to translation, nucleotide metabolism, cofactors and vitamin metabolism, and replication and repair were positively correlated with samples from HIV-positive patients (Fig. S4E). Pathways related to membrane transport, cellular processes and signaling, lipid and carbohydrate metabolism, xenobiotics biodegradation, and metabolism and transcription were positively correlated with samples from HIV-negative patients (Fig. S4E).

**FIG 6:**
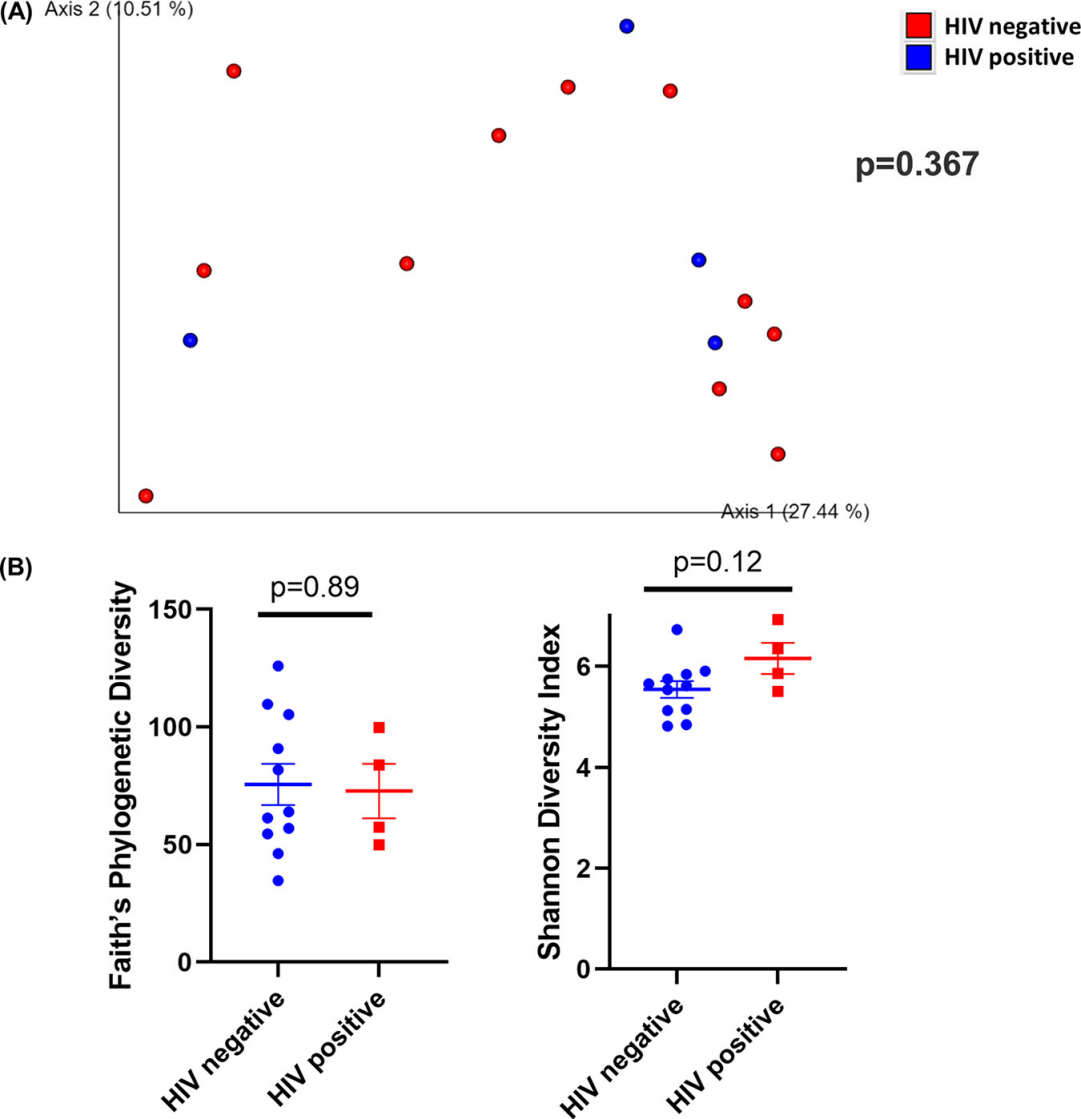
Diversity analysis of the TI brush samples. (A) Principal-coordinate analysis (PCoA) plot of weighted UniFrac distances (metrics of β-diversity). Samples grouped by HIV-negative (*n* = 11) and -positive (*n* = 4); *P* = 0.367. (B) Faith’s phylogenic diversity and Shannon diversity index (metrics of α-diversity) at a sequencing depth of 80,000 reads. Samples were grouped by HIV-negative (*n* = 11) and -positive (*n* = 4) status. Error bars represent SEM.

In TI brush samples, both HIV-positive and HIV-negative samples were not significantly different (*P* = 0.367) as assessed by weighted UniFrac distances (Fig. 6A). Additionally, there was no difference in Faith’s phylogenetic diversity (*P* = 0.89) or the Shannon diversity index (*P* = 0.12) between TI wash samples between groups (Fig. 6B).

Moreover, LEfSe analysis showed no bacteria taxa enriched in either group. TI brush samples from HIV-positive patients were not significantly different from HIV-negative patients in biofilm-forming bacteria (*P* = 0.75), pathogenic bacteria (*P* = 0.22), or Gram-positive (*P* = 0.41) or Gram-negative bacteria (*P* = 0.41) (Fig. S5A to D). Pathways related to replication and repair, translation, nucleotide metabolism, and cofactors and vitamin metabolism were positively correlated with samples from HIV-positive patients (Fig. S5E). Pathways related to membrane transport, cellular processes and signaling, carbohydrate metabolism, xenobiotics biodegradation, and metabolism and transcription were positively correlated with samples from HIV-negative patients (Fig. S5E).

To investigate the impact of sampling sites on the microbiome, we first analyzed samples from HIV-negative patients alone. TI brush samples significantly clustered apart from the three other sampling locations (TI wash, colon wash, and colon brush) in the PCoA plot (false-discovery rate–adjusted *P* value [*q*] = 0.001) (Fig. S7A). Additionally, both TI and colon brush samples had higher Faith’s phylogenetic diversity than TI and colon wash samples (*P* < 0.001) (Fig. S7B), indicating more unique bacteria taxa are present on the intestinal epithelium. Additionally, there was no significance (*P* = 0.9) between TI and colon wash samples assessed by Faith’s phylogenetic diversity.

However, when we analyzed samples from HIV-positive patients alone, we did not observe separation in the PCoA plot between any gut samples (TI wash, TI brush, colon wash, and colon brush) (*q* > 0.05) (Fig. S8A). However, TI brush exhibited a trend (*q* = 0.07) toward higher Faith’s phylogenetic diversity than TI and colon wash samples (Fig. S8B).

### The oral microbiome is not altered in patients with HIV on ART.

**FIG 7:**
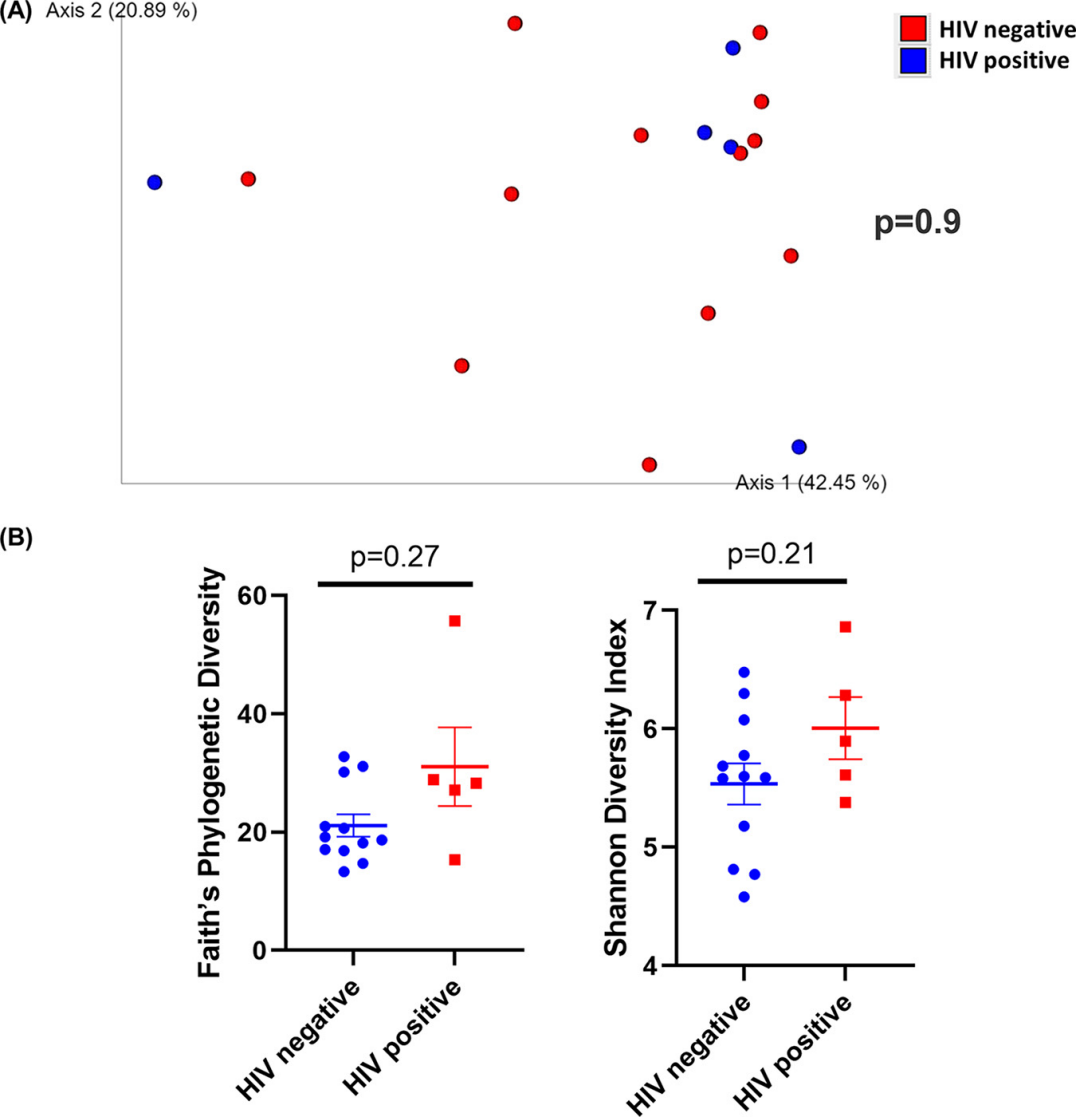
Diversity analysis of the saliva samples. (A) Principal-coordinate analysis (PCoA) plot of weighted UniFrac distances (metrics of β-diversity). Samples grouped by HIV-negative (*n* = 12) and -positive (*n* = 5) status; *P* = 0.9. (B) Faith’s phylogenic diversity and Shannon diversity index (metrics of α-diversity) at a sequencing depth of 80,000 reads. Samples grouped by HIV-negative (*n* = 12) and -positive (*n* = 5) status. Error bars represent SEM.

Among all oral samples, 1,520 unique ASVs were identified (Table S3). The composition of the oral microbiome was plotted at the phylum and genus levels and grouped by HIV status (Fig. S6). Our results indicated that the oral microbiome is not altered with HIV infection. When β-diversity was measured using weighted UniFrac distances and visualized with PCoA plots, salivary samples from patients with HIV did not cluster apart from salivary samples from patients without HIV (*P* = 0.9) (Fig. 7A). Furthermore, neither Faith’s phylogenetic diversity (*P* = 0.27) nor Shannon diversity index (*P* = 0.21) demonstrated differences between HIV-positive and HIV-negative saliva samples (Fig. 7B). Notably, the PCoA plot of HIV-negative and positive samples also confirmed that the oral microbiome was significantly different from the intestinal microbiome (*q* < 0.01) (Fig. S7 and S8). Oral samples from HIV-positive patients were not significantly different from HIV-negative patients in biofilm-forming bacteria (*P* = 0.15), pathogenic bacteria (*P* = 0.5), Gram-positive (*P* = 0.38), or Gram-negative bacteria (*P* = 0.38) (data not shown). No KEGG level 2 pathways were significantly different between groups in oral samples (*P* > 0.05) (data not shown).

### The microbiome does not cluster by patients.

To determine if samples collected from a given individual were clustered within the subject, we grouped samples by individuals and performed a diversity analysis. Analysis of β-diversity using weighted UniFrac distances showed that the samples were not clustered by each individual, with no significant difference between any individuals (*q* > 0.05) (Fig. S9).

## DISCUSSION

In this study, we characterized both the oral and intestinal microbiome using multiple sampling sites (colon brush, colon wash, TI brush, and TI wash) in patients with HIV, which has not previously been described in samples from the same patients. Here, we report alterations in the composition and predicted function of the intestinal microbiome in HIV-infected patients on ART compared to uninfected individuals. Interestingly, we found no differences in the composition and predicted function of the oral microbiome between groups. In our study, we also compared the intestinal microbiome by sampling site and demonstrated site-specific alterations in the microbiome, with the microbiome unaltered in TI brush samples, while other sites showed significant differences.

Our findings regarding the β-diversity in intestinal samples of HIV patients on ART are largely consistent with previous studies (9, 10, 31), which have also demonstrated a shift in the overall intestinal microbial community. It is worth noting that in some of these studies, HIV-positive patients were viremic untreated, which could be a confounding factor. However, a shift in β-diversity has been shown in both HIV-infected untreated and ART-treated populations. McHardy et al. reported that HIV-untreated subjects were significantly different from healthy controls, whereas ART-treated subjects were not statistically different from healthy controls (14). Mutlu et al. confirmed that samples from HIV-infected and untreated subjects clustered differently than those from controls using both UniFrac and Bray-Curtis metrics (26). In terms of α-diversity, this study did not find a significant difference in Faith’s phylogenetic diversity; however, a significant increase in Shannon diversity index was observed. Consistently, Vujkovic-Cvijin et al. reported no changes in community richness or evenness between HIV-infected (untreated and on ART) and uninfected subjects in the community (15). Dillon et al. observed similar sample richness and evenness between uninfected and HIV-infected untreated subjects and a trend toward greater evenness in HIV-infected individuals (29). However, other studies have reported either an increase or decrease in α-diversity in HIV patients (untreated and on ART) (13, 14, 32, 33). Additionally, Lozupone et al. reported that untreated HIV patients had higher α-diversity than ART-treated and HIV-negative individuals (34). The discrepancies in results are likely due to variations in diet associated with different regions and ethnicities, relatively small sample sizes in each study, and lack of proper control subjects.

We also identified differentially enriched bacteria with HIV infection by sampling site. Several studies (29, 34, 35) have reported enrichment of *Prevotella* in HIV-positive subjects (ART treated and untreated), which is in agreement with our study showing that *Prevotella* had the highest LDA score in LEfSe analysis. Interestingly, one study reported that *Prevotella* was significantly decreased after ART, suggesting its involvement during HIV inflammation (13). Furthermore, Noguera-Julian et al. observed that the balance between *Prevotella* and *Bacteroides* may be correlated with sexual preferences rather than HIV *per se* (36). Specifically, they found that men who have sex with men (MSM) had increased *Prevotella*, whereas most non-MSM subjects were enriched in *Bacteroides*, regardless of HIV-1 status. Notably, many of our identified bacteria enriched in HIV patients were consistent with previous studies. McHardy et al. reported *Fusobacteria* and *Peptostreptococcus* among the bacteria that were significantly enriched in HIV-positive patients (14). Lozupone et al. also reported that *Peptococcus*, Mitsuokella jalaludinii, and Megasphaera elsdenii increased in relative abundance in patients with HIV infection (untreated and on ART) (34, 37). Mutlu et al. observed an increase in *Mogibacterium* and unclassified *Fusobacteriaceae* associated with HIV patients on ART (26). However, there was discordance in our studies and others regarding *Eubacterium*, with two studies reporting that *Eubacterium* was depleted in HIV-associated mucosal samples (14, 26). Discrepancies could be attributed to the method for sample collection and diet effects associated with different regions and ethnicities. Still, the bacteria genera we identified that were more enriched in non-HIV patients are consistent with previous literature. For example, McHardy et al. and Mutlu et al. reported that *Ruminococcus* was depleted in HIV-infected subjects (untreated and on ART) (14, 26), which is in accordance with our finding that *Ruminococcus* is more abundant in HIV-negative patients. Consistently, others have reported genus *Blautia* was decreased in HIV patients (untreated and on ART) in accordance with our own observations (26, 29).

Previous studies also examined functional shifts in the intestinal metagenome in HIV-infected individuals. McHardy et al. reported that the imputed microbial metagenome from HIV patients without ART treatment was depleted of amino acids metabolism, CoA biosynthesis, and fructose/mannose metabolism, and it was enriched for glutathione metabolism, selenocompound metabolism, folate biosynthesis, and siderophore biosynthesis (14). Additionally, Vázquez-Castellanos et al. did metagenome sequencing on the intestinal microbiota and found enrichment of genes involved in various pathogenic processes, lipopolysaccharide biosynthesis, bacterial translocation, and other inflammatory pathways in HIV-positive individuals on ART. Furthermore, genes involved in amino acid metabolism and energy processes were depleted in HIV-positive individuals (1). Our own observations of altered microbiome functions largely agreed with previous studies. BugBase and PICRUSt functional algorithms predicted that potentially pathogenic bacteria and Gram-negative bacteria were enriched in HIV-positive patients. Similarly, our functional profiling predicted depletion of carbohydrates and lipids metabolism in HIV-positive individuals, which are both related to overall energy metabolism. However, we did not observe significant changes in amino acid metabolism in our study, which could be partially attributed to the diet of the study subjects.

To date, very few studies have investigated changes in the intestinal microbiome using multiple sampling sites. One study concluded that fecal aspirates and stool samples generally represent the same pattern of bacteria from *Bacteroidetes* in mucosa-adherent bacteria, but HIV-1-associated changes in *Proteobacteria* and *Firmicutes* were mucosa specific (29). Furthermore, Yang et al. found that the difference between HIV-positive (untreated and on ART) and -negative groups was not significant when all four body sites (mouth, esophagus, stomach, and duodenum) were included, but the difference became significant when the proximal intestinal (esophagus, stomach, and duodenum) was analyzed. In site-specific analyses, the separation between HIV patients and controls was only significant in the duodenum but not significant in each of the three other body sites examined (38). Mutlu et al. reported that all sample types (terminal ileum, right colon, left colon, and feces) had fewer operational taxonomic units (OTUs) in the HIV group on ART than in healthy controls. Additionally, they observed the separation of samples for each sample type when β-diversity was measured by UniFrac metric. The dispersion of samples was visually more apparent for the terminal ileum and right colon samples and less overlapping in the left colon and fecal samples (26). Our study also reflects sampling-site-specific variations in the intestinal microbiome. Samples from colon wash, colon brush, and TI wash were significantly different in microbial composition between HIV-infected and uninfected subjects, while samples from TI brush and saliva were not significant. Moreover, we demonstrated that TI brush samples were very different from the three other sampling locations in HIV-negative patients (TI wash, colon wash, and colon brush) in the β-diversity plot (see Fig. S7 in the supplemental material). Our study also highlighted differences in α-diversity in sampling site (lumen wash versus lumen brush), as both TI and colon brush samples had higher Faith’s phylogenetic diversity than TI and colon wash samples (Fig. S7), indicating more unique bacteria taxa are present on the intestinal epithelium. Overall, our results suggested that HIV infection affected the microbiome in a site-specific manner.

Previous studies have also profiled the oral microbiome with HIV infection. However, there have been inconsistent reports on the effect on α-diversity. For instance, one group reported that the oral microbiota in HIV-infected patients (untreated and on ART) had higher α-diversity as well as higher bacterial loads (19). However, others reported a lower oral microbiome richness in HIV-infected individuals on ART (21, 39). The increase in α-diversity could be the result of an increase in pathogen colonization, and the decrease could be the result of a few pathogens that dominated the oral environment. Furthermore, a range of secretory antimicrobial peptides could also play a significant role in the balance (40). That being said, while several studies have demonstrated that the oral microbiome composition changes during HIV infection (untreated and on ART) (16, 39), other studies have reported no major change in the oral microbiome or attributed changes to comorbid periodontal disease (21, 23, 41). In the current study, we did not observe any significant change in oral microbiome richness, evenness, composition, or predicted functions.

Collectively, our study highlighted site-specific alterations in the microbiome and supported the possibility of targeting certain regions in the gastrointestinal tract to mitigate dysbiosis in HIV-positive patients on ART. Additionally, the use of brush and wash samples allowed us to determine changes in adherent or loosely associated mucosa microbiota. Broadly, our findings add to the general knowledge to aid the development of precise location and microbial targeted interventions. This study also had several limitations, which must be noted. First, this study had a small sample size (HIV positive, 5 individuals; HIV negative, 12 individuals), reflecting the difficulty in recruiting subjects for biopsy and/or poor adherence with patients’ follow-up visits for saliva samples. A larger sampling size would have permitted more differentially abundant bacteria taxa to be detected. Next, this study did not correlate microbiome findings with immune measurements. Importantly, the correlation of CD4^+^ T cells has often been associated with specific bacteria taxa, and these associations were lacking in this study. Moreover, the vast majority of the subjects in our study were obese, which was known to have independent effects on the microbiome. Enrollment of more subjects within the normal body mass index (BMI) range could have increased the representativeness of the current study.

As this study collected samples from biopsy rather than stool, microbiome analyses presented here are less biased by the substantial variations caused by food/ingested materials in human subjects. Still, it must be noted that some studies investigating the intestinal and oral microbiome with HIV have been inconsistent in their metrics, which have largely been attributed to diet. For instance, some studies have reported patients with HIV had lower α-diversity (13, 14, 26) in intestinal samples; however, others have claimed HIV patients had higher (34) or no change in intestinal α-diversity (15, 29). Similarly, there have been differences in enriched bacterial taxa across studies in intestinal samples in HIV patients. Some groups reported depletion of *Bacteroides* and enrichment of *Prevotella* in HIV (26, 34). Others reported depletion of *Clostridiales* in untreated HIV patients (14). Furthermore, increased *Proteobacteria* and decreased *Firmicutes* in HIV have also been reported (38). Additionally, there have been inconsistencies in the reporting of the oral microbiome between HIV-infected patients and healthy controls (42). Jiménez-Hernández et al. reported that the salivary α-diversity in HIV-infected individuals was significantly higher than those in HIV-uninfected samples (19). Others revealed that the oral α-diversity in patients with HIV was significantly lower than the uninfected individuals (16, 21, 39). Thus, we consider our approach using biopsy specimens to profile the mucosal microbiota at specific sites within the gastrointestinal tract and saliva samples to characterize the oral microbiome more advantageous than these aforementioned studies.

In conclusion, in the current study, we showed altered intestinal microbiome composition and function in patients with HIV on ART, with no significant differences in the oral microbiome between HIV-infected and uninfected patients. Here, we also characterized changes in the intestinal microbiome by sampling site and found that samples from colon wash, colon brush, and TI wash were significant between groups, while samples from TI brush and saliva were not significant. As the role of the microbiota is becoming increasingly clear in HIV infection, our study, which profiled the oral microbiota, strongly adherent mucosal communities (brush), and loosely mucosa-associated microbiota (wash) helps put into context site-specific changes with HIV infection.

## MATERIALS AND METHODS

### Study subjects and sample collection.

Inclusion criteria were individuals who were 18 years and older who consented to this study and were scheduled to undergo endoscopy and/or colonoscopy or sigmoidoscopy at either the University of Miami Hospital or the University of Miami Hospital & Clinics/Sylvester Comprehensive Cancer Center. HIV-positive patients were defined as having a previous diagnosis of HIV per electronic medical record. Exclusion criteria were patients who had not agreed to participate and had not signed the consent form; patients with diagnosis of irritable bowel syndrome, inflammatory bowel disease, and colon cancer; and patients on antibiotics or steroids within 30 days before visits. Patients provided >5 mL saliva during hospital visits, using the Omnigene oral kit (DNA Genotek; catalog no. OM-501). TI brush and colon brush were collected during colonoscopy or sigmoidoscopy with a sheathed cytology brush (ConMed; catalog no. 000110). TI wash and colon wash were collected during colonoscopy or sigmoidoscopy, with sterile saline flushing the TI or colon when the microscope reached the target compartment. All samples were stored at −80°C until processing.

### DNA extraction and 16S rRNA gene sequencing.

For intestinal samples (colon brush, colon wash, TI brush, and TI wash), DNA was isolated using DNeasy PowerSoil Pro kit (Qiagen; catalog no. 47016). For saliva samples, DNA was isolated using QIAamp DNA blood minikit (Qiagen; catalog no. 51104). During DNA extraction, two extraction controls in each batch were included to remove potential contamination from kit reagents. Sequencing was performed by the University of Minnesota Genomics Center. The hypervariable V4 region of the 16S rRNA gene was PCR amplified using the forward primer 515F (GTGCCAFCMGCCGCGGTAA), reverse primer 806R (GGACTACHVGGGTWTCTAAT), Illumina adaptors, and molecular barcodes to produce 427-bp amplicons. Amplicons were sequenced with the Illumina MiSeq version 3 platform, generating 300-bp paired-end reads. The extraction controls could not be PCR amplified due to very low copy number (less than 10 in extraction control versus 10e-8 copies in experimental samples) and were therefore excluded from the sequencing process.

### Bioinformatics analysis.

Demultiplexed sequence reads were clustered into amplicon sequence variants (ASVs) with the DADA2 package (version 1.21.0) (43) implemented in R (version 4.0.3) and RStudio (version 1.1.463). The steps of the DADA2 pipeline include error filtering, trimming, learning of error rates, denoising, merging of paired reads, and removal of chimeras. On average, 178,608 sequence reads per intestinal sample and 176,926 sequence reads per saliva sample were kept after error filtering and other steps (see Table S2 in the supplemental material). During trimming, the forward and reverse reads were truncated at positions 230 and 180 to remove low-quality tails. The ASV table generated by DADA2 was imported into the QIIME2 pipeline (44) for diversity analyses and taxonomic assignment. Diversity analyses were performed by using the QIIME diversity core-metrics-phylogenetic script with a sampling depth of 80,000. Taxonomic assignment of ASVs was done to the genus level using a naive Bayesian classifier (45) implemented in QIIME2 with the Greengenes reference database (13_8 99%) (46). MicrobiomeAnalyst (47) was used for generating bar plots and linear discriminant analysis effect size (LEfSe)(48) plot. The threshold on the logarithmic LDA score for discriminative features was set to 2. The cutoff for false-discovery rate–adjusted *P* value (*q* value) was set to 0.1 for LEfSe analysis.

PICRUSt (49) is a computational approach to predict the functional composition of a metagenome using 16S data with reference genomes from Greengenes (46) and IMG (50) databases. PICRUSt pathway prediction was implemented within the galaxy app (https://huttenhower.sph.harvard.edu/galaxy/). KEGG Orthology (51) was used to predict the metagenome. The KEGG pathway was categorized into pathway hierarchy level 2. STAMP (52) was used for identifying pathways that were differentially abundant and for generating extended error bar plots. BugBase (53) is a microbiome analysis algorithm that predicts high-level phenotypes present in microbiome samples using 16S amplicon data. The BugBase phenotype predictions were implemented using the online web app (https://bugbase.cs.umn.edu/).

### Statistical analysis.

Mann-Whitney test or Kruskal-Wallis test was used to detect if α-diversity differed across treatments. Permutational multivariate analysis of variance (PERMANOVA) was used to detect if β-diversity differed across treatments. The Benjamini-Hochberg method was used for controlling the false-discovery rate (*q* value). A *P* value of <0.05 was considered to be statistically significant.

### Ethics statement.

The study protocol was approved by The University of Miami Institutional Review Board (20160338). Written and informed consent was obtained from each patient before enrollment. All patients were enrolled at the University of Miami Hospital.

### Data availability.

Sequence data are available at the BioStudies database (54) (https://www.ebi.ac.uk/biostudies/) under accession number S-BSST836.
