# Supplementary material for: HIV-Positive Patients on Antiretroviral Therapy Have an Altered Mucosal Intestinal but Not Oral Microbiome
Source: Microbiol Spectr. 2022 Dec 13;11(1):e02472-22. doi: 10.1128/spectrum.02472-22 (PMC9927552; doi:10.1128/spectrum.02472-22)
Supplement: Supplemental file 1 — Supplemental material. Download spectrum.02472-22-s0001.pdf, PDF file, 3.8 MB [file spectrum.02472-22-s0001.pdf]

Supporting Information

Fig S1. Bar plots of bacterial composition in the intestinal samples. Samples were grouped by HIV-negative (n=44) and positive (n=18). (A) at the phylum level. (B) at the genus level.

Figure S2. Predictive functional analysis of colon wash samples. (A) BugBase predicted the relative abundance of biofilm-forming bacteria. Samples grouped by HIV-negative (n=12) and positive (n=5), p-value = 0.16. (B) BugBase predicted relative abundance of potentially pathogenic bacteria. Samples grouped by HIV-negative (n=12) and positive (n=5), p-value = 0.06. (C) BugBase predicted relative abundance of Gram-positive bacteria. Samples grouped by HIV-negative (n=12) and positive (n=5), p-value = 0.02. (D) BugBase predicted relative abundance of Gram-negative bacteria. Samples grouped by HIV-negative (n=12) and positive (n=5), p-value = 0.02. (E) The KEGG pathway of gut microbiota was predicted using PICRUSt (Phylogenetic Investigation of Communities by Reconstruction of Unobserved States). Data are presented in a bar plot with 95% confidence intervals and p-values between gut samples from HIV-positive and negative patients.

Figure S3. Predictive functional analysis of colon brush samples. (A) BugBase predicted the relative abundance of biofilm-forming bacteria. Samples grouped by HIV-negative (n=11) and positive (n=5), p-value = 0.11. (B) BugBase predicted relative abundance of potentially pathogenic bacteria. Samples grouped by HIV-negative (n=11) and positive (n=5), p-value = 0.008. (C) BugBase predicted relative abundance of Gram-positive bacteria. Samples grouped by HIV-negative (n=11) and positive (n=5), p-value = 0.44. (D) BugBase predicted relative abundance of Gram-negative bacteria. Samples grouped by HIV-negative (n=11) and positive (n=5), p-value = 0.44. (E) The KEGG pathway of gut microbiota was predicted using PICRUSt (Phylogenetic Investigation of Communities by Reconstruction of Unobserved States). Data are presented in a bar plot with 95% confidence intervals and p-values between gut samples from HIV-positive and negative patients.

Figure S4. Predictive functional analysis of TI wash samples. (A) BugBase predicted the relative abundance of biofilm-forming bacteria. Samples grouped by HIV-negative

(n=10) and positive (n=4), p-value = 0.14. (B) BugBase predicted relative abundance of potentially pathogenic bacteria. Samples grouped by HIV-negative (n=10) and positive (n=4), p-value = 0.04. (C) BugBase predicted relative abundance of Gram-positive bacteria. Samples grouped by HIV-negative (n=10) and positive (n=4), p-value = 0.04. (D) BugBase predicted relative abundance of Gram-negative bacteria. Samples grouped by HIV-negative (n=10) and positive (n=4), p-value = 0.04. (E) The KEGG pathway of gut microbiota was predicted using PICRUSt (Phylogenetic Investigation of Communities by Reconstruction of Unobserved States). Data are presented in a bar plot with 95% confidence intervals and p-values between gut samples from HIV-positive and negative patients.

Figure S5. Predictive functional analysis of TI brush samples. (A) BugBase predicted the relative abundance of biofilm-forming bacteria. Samples grouped by HIV-negative (n=11) and positive (n=4), p-value = 0.75. (B) BugBase predicted relative abundance of potentially pathogenic bacteria. Samples grouped by HIV-negative (n=11) and positive (n=4), p-value = 0.22. (C) BugBase predicted relative abundance of Gram-positive bacteria. Samples grouped by HIV-negative (n=11) and positive (n=4), p-value = 0.41. (D) BugBase predicted relative abundance of Gram-negative bacteria. Samples grouped by HIV-negative (n=11) and positive (n=4), p-value = 0.41. (E) The KEGG pathway of gut microbiota was predicted using PICRUSt (Phylogenetic Investigation of Communities by Reconstruction of Unobserved States). Data are presented in a bar plot with 95% confidence intervals and p-values between gut samples from HIV-positive and negative patients.

Figure S6. Bar plots of bacterial composition in the saliva samples. Samples were grouped by HIV-negative (n=12) and positive (n=5). (A) at the phylum level. (B) at the genus level.

Figure S7. Diversity analysis of the HIV-negative samples by sampling site. (A) Principal coordinates analysis (PCoA) plot of weighted UniFrac distances (metrics of β-diversity). Samples grouped by TI wash (n=10), TI brush (n=11), colon wash (n=12), colon brush (n=11), and saliva (n=12). False discovery rate corrected q-value < 0.001

between saliva and every other group. q-value = 0.001 between TI brush and other intestinal samples (TI wash, colon wash, colon brush). q-value > 0.2 between colon, colon brush, and TI wash. (B) Faith's Phylogenic Diversity (metrics of α-diversity) at sequencing depth 80,000. Samples grouped by TI wash (n=10), TI brush (n=11), colon wash (n=12), colon brush (n=11), and saliva (n=12).

Figure S8. Diversity analysis of the HIV-positive samples sampling site. (A). Principal coordinates analysis (PCoA) plot of weighted UniFrac distances (metrics of β-diversity). Samples grouped by TI wash (n=4), TI brush (n=4), colon wash (n=5), colon brush (n=5), and saliva (n=5). False discovery rate corrected q-value < 0.001 between saliva and every other group. q-value > 0.05 between colon, colon brush, TI brush, and TI wash samples. (B). Faith's Phylogenic Diversity (metrics of α-diversity) at sequencing depth 80,000. Samples grouped by TI wash (n=4), TI brush (n=4), colon wash (n=5), colon brush (n=5), and saliva (n=5). There was no significance between any groups (q>0.05).

Figure S9. Diversity analysis of all subjects by individual patients. Principal coordinates analysis (PCoA) plot of weighted UniFrac distances (metrics of β-diversity). There was no significance between any groups (q>0.05).

Table S1. Patients' clinical characteristics.

Table S2. Sequencing count of samples through each step in the DADA2 pipeline.

Table S3. Sequencing count of amplicon sequence variants (ASVs) in all sample types.

Table S4. Predicted pathway by PICRUSt.

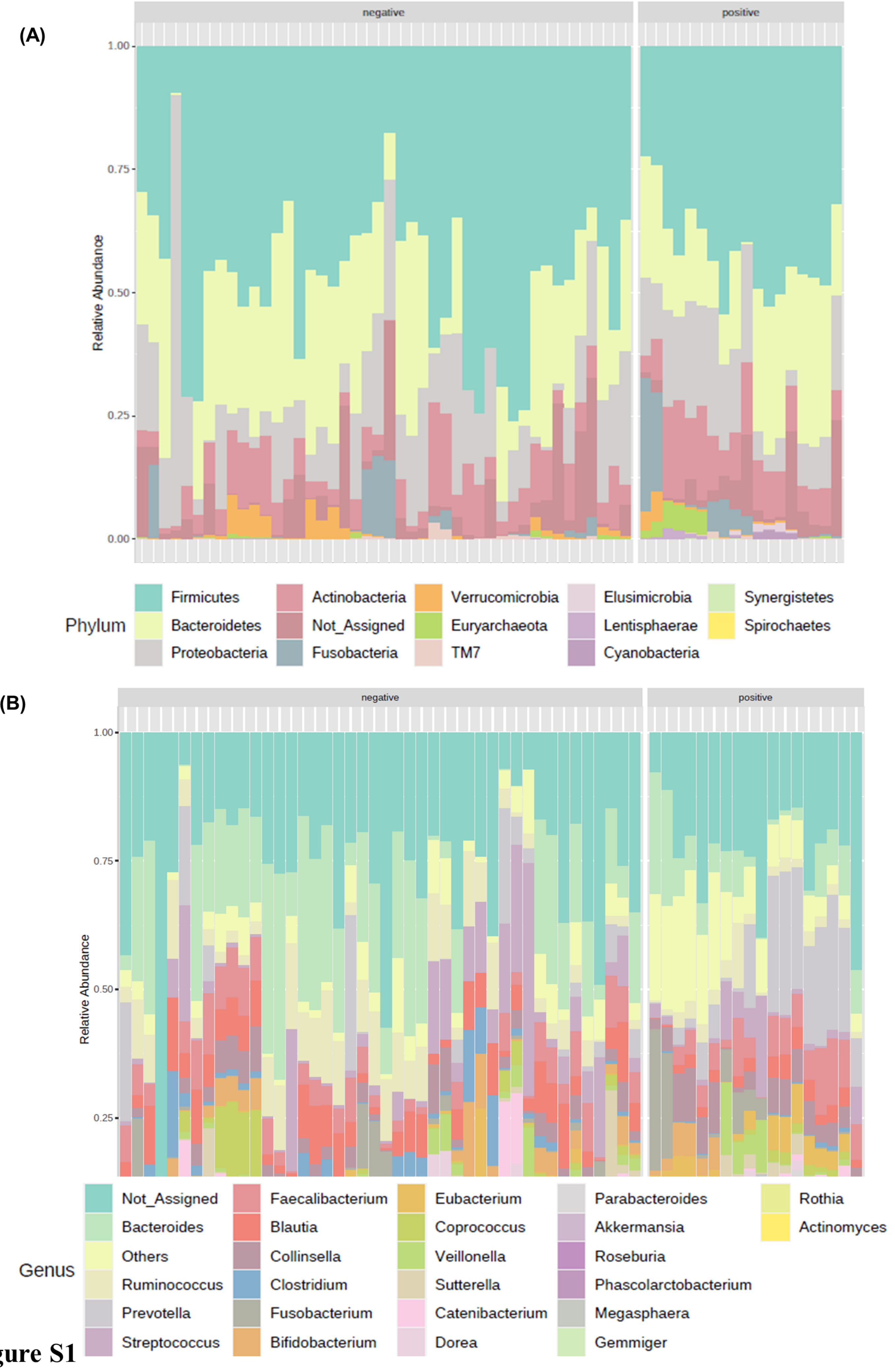
(A)
negative
positive
1.00
0.75
0.50
0.25
0.00
Relative Abundance
Phylum
Firmicutes
Bacteroidetes
Proteobacteria
Actinobacteria
Not_Assigned
Fusobacteria
Verrucomicrobia
Euryarchaeota
TM7
Elusimicrobia
Lentisphaerae
Cyanobacteria
Synergistetes
Spirochaetes
(B)
negative
positive
1.00
0.75
0.50
0.25
Relative Abundance
Genus
Not_Assigned
Bacteroides
Others
Ruminococcus
Prevotella
Streptococcus
Faecalibacterium
Blautia
Collinsella
Clostridium
Fusobacterium
Bifidobacterium
Eubacterium
Coprococcus
Veillonella
Sutterella
Catenibacterium
Dorea
Parabacteroides
Akkermansia
Roseburia
Phascolarctobacterium
Megasphaera
Gemmiger
Rothia
Actinomyces

Figure S1

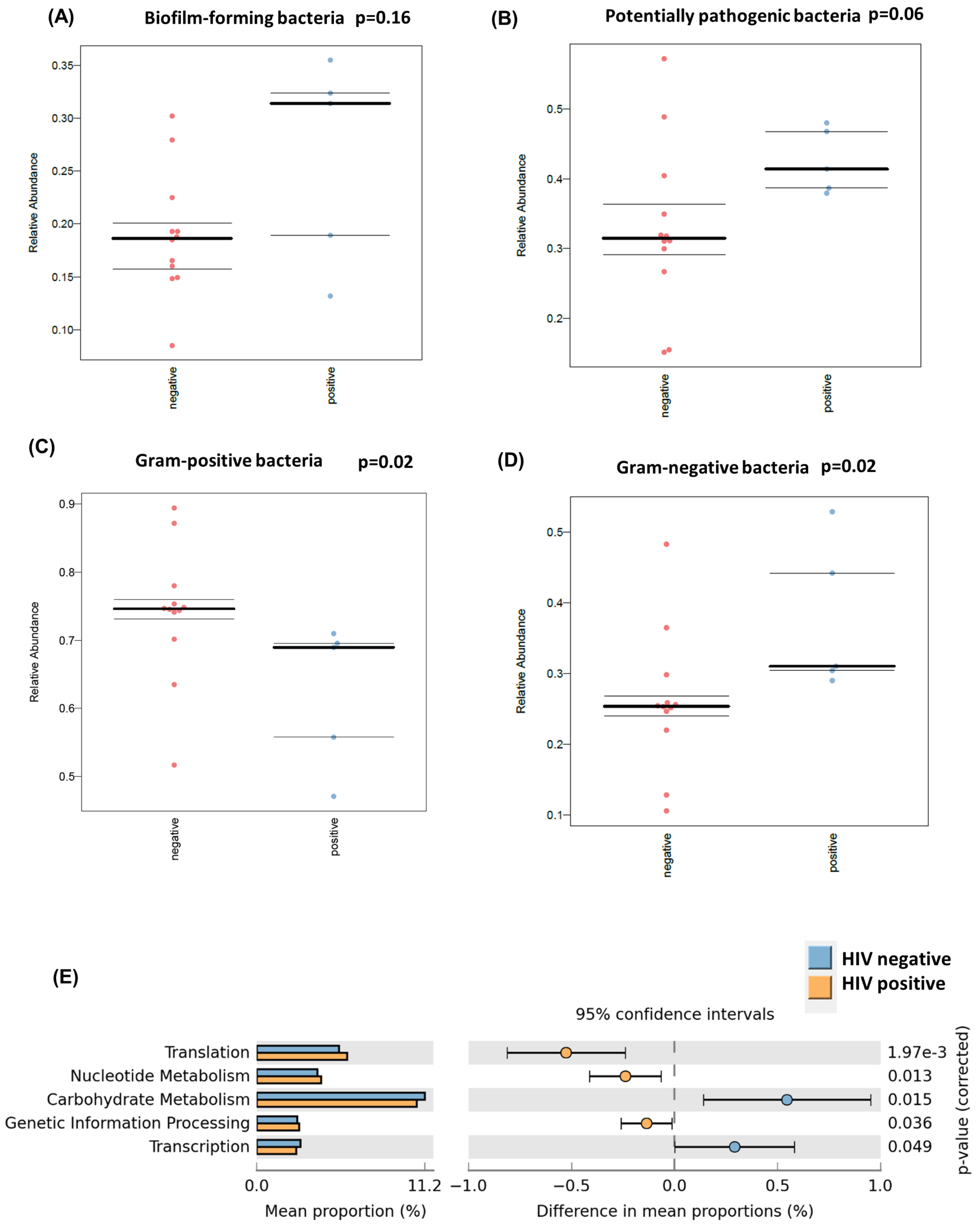

(A)
Biofilm-forming bacteria p=0.16
Relative Abundance
0.35
0.30
0.25
0.20
0.15
0.10
negative
positive
(B)
Potentially pathogenic bacteria p=0.06
Relative Abundance
0.5
0.4
0.3
0.2
negative
positive
(C)
Gram-positive bacteria p=0.02
Relative Abundance
0.9
0.8
0.7
0.6
0.5
negative
positive
(D)
Gram-negative bacteria p=0.02
Relative Abundance
0.5
0.4
0.3
0.2
0.1
negative
positive
(E)
HIV negative
HIV positive
95% confidence intervals
Translation
Nucleotide Metabolism
Carbohydrate Metabolism
Genetic Information Processing
Transcription
1.97e-3
0.013
0.015
0.036
0.049
p-value (corrected)
0.0
11.2
Mean proportion (%)
−1.0
−0.5
0.0
0.5
1.0
Difference in mean proportions (%)

Figure S2

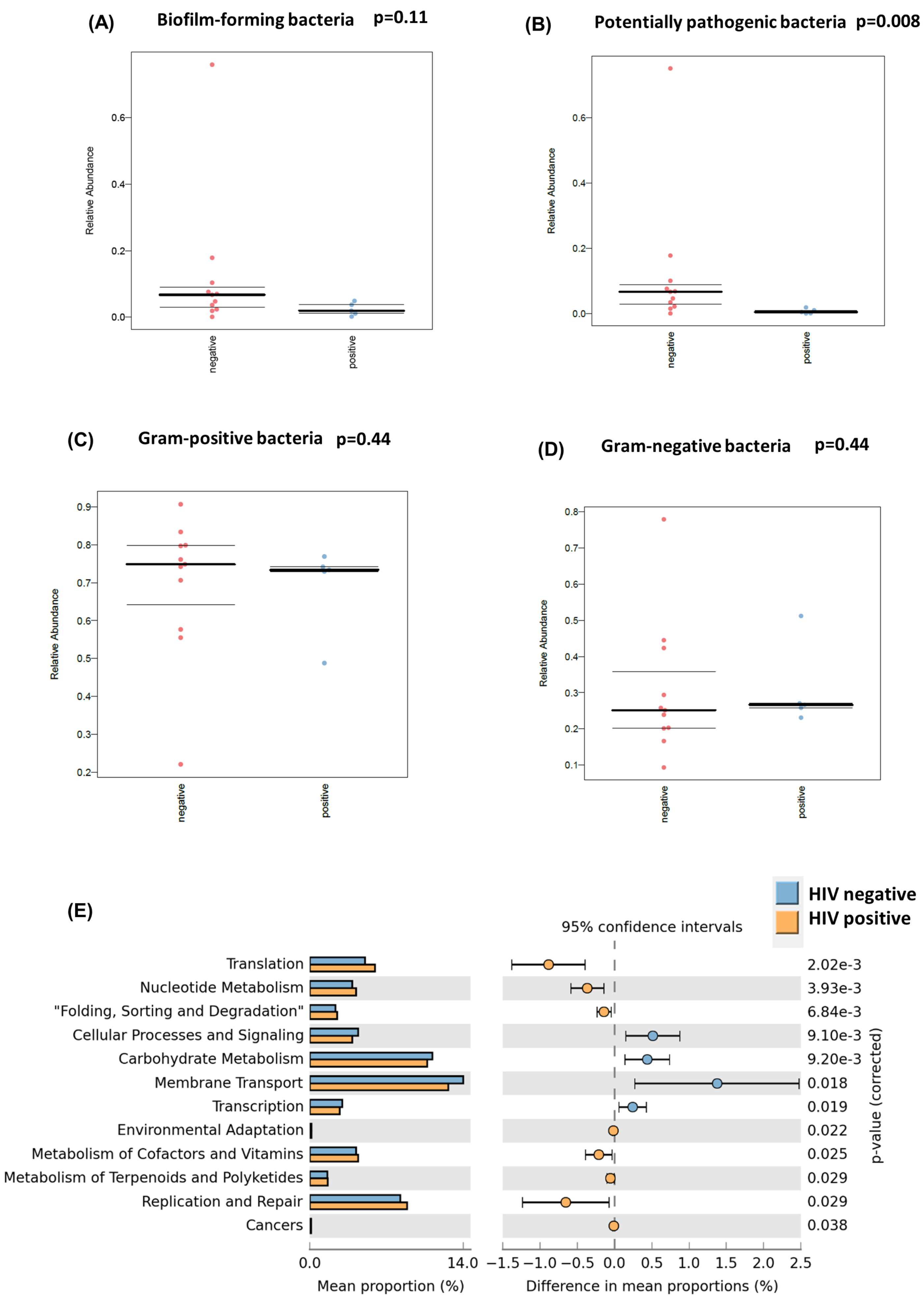
(A)
Biofilm-forming bacteria p=0.11
Relative Abundance
0.0
0.2
0.4
0.6
negative
positive
(B)
Potentially pathogenic bacteria p=0.008
Relative Abundance
0.0
0.2
0.4
0.6
negative
positive
(C)
Gram-positive bacteria p=0.44
Relative Abundance
0.2
0.3
0.4
0.5
0.6
0.7
0.8
0.9
negative
positive
(D)
Gram-negative bacteria p=0.44
Relative Abundance
0.1
0.2
0.3
0.4
0.5
0.6
0.7
0.8
negative
positive
(E)
HIV negative
HIV positive
95% confidence intervals
Translation
Nucleotide Metabolism
"Folding, Sorting and Degradation"
Cellular Processes and Signaling
Carbohydrate Metabolism
Membrane Transport
Transcription
Environmental Adaptation
Metabolism of Cofactors and Vitamins
Metabolism of Terpenoids and Polyketides
Replication and Repair
Cancers
2.02e-3
3.93e-3
6.84e-3
9.10e-3
9.20e-3
0.018
0.019
0.022
0.025
0.029
0.029
0.038
p-value (corrected)
0.0
14.0
Mean proportion (%)
−1.5 −1.0 −0.5 0.0 0.5 1.0 1.5 2.0 2.5
Difference in mean proportions (%)

Figure S3

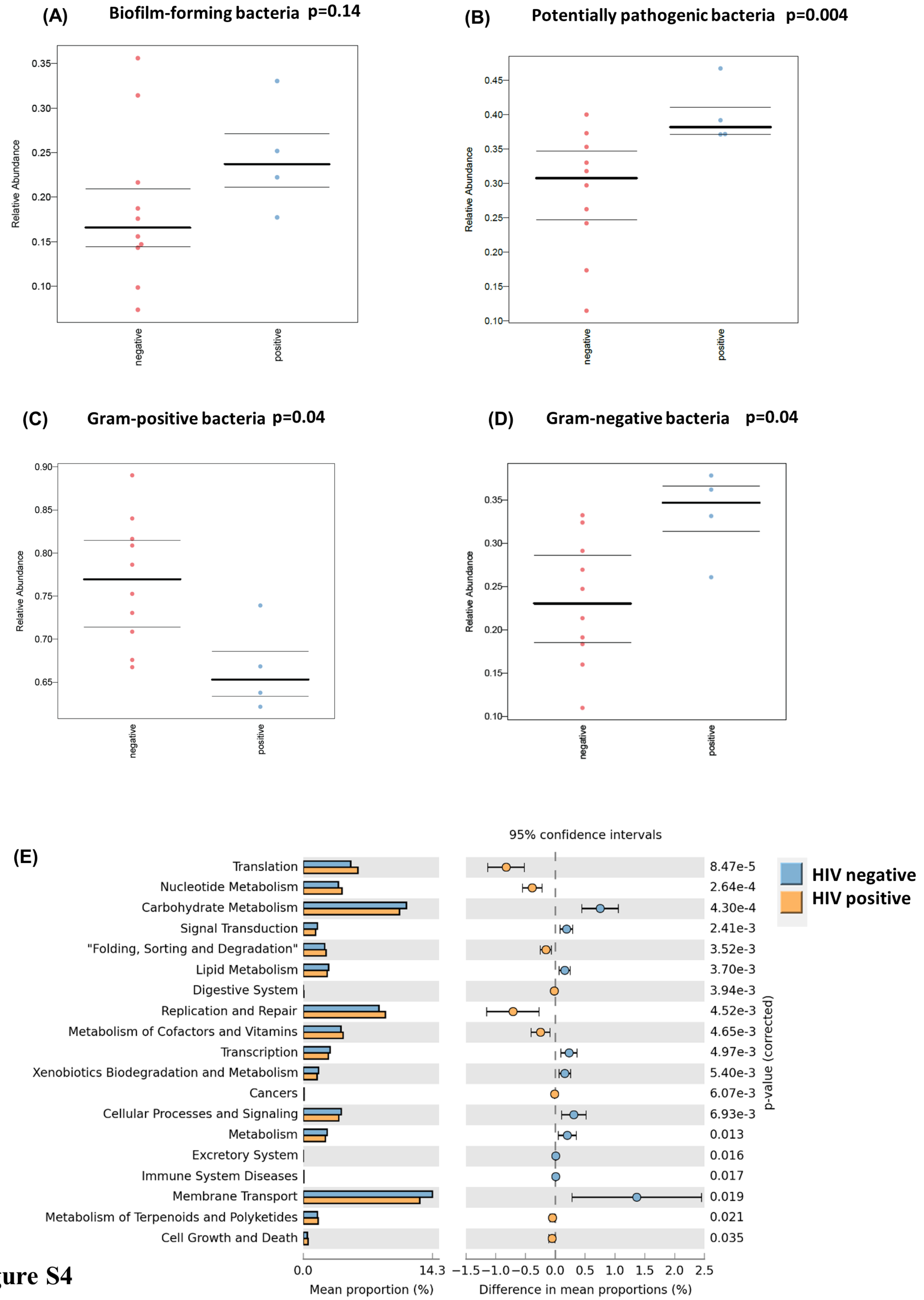
(A)
Biofilm-forming bacteria p=0.14
Relative Abundance
0.35
0.30
0.25
0.20
0.15
0.10
negative
positive
(B)
Potentially pathogenic bacteria p=0.004
Relative Abundance
0.45
0.40
0.35
0.30
0.25
0.20
0.15
0.10
negative
positive
(C)
Gram-positive bacteria p=0.04
Relative Abundance
0.90
0.85
0.80
0.75
0.70
0.65
negative
positive
(D)
Gram-negative bacteria p=0.04
Relative Abundance
0.35
0.30
0.25
0.20
0.15
0.10
negative
positive
(E)
95% confidence intervals
Translation
Nucleotide Metabolism
Carbohydrate Metabolism
Signal Transduction
"Folding, Sorting and Degradation"
Lipid Metabolism
Digestive System
Replication and Repair
Metabolism of Cofactors and Vitamins
Transcription
Xenobiotics Biodegradation and Metabolism
Cancers
Cellular Processes and Signaling
Metabolism
Excretory System
Immune System Diseases
Membrane Transport
Metabolism of Terpenoids and Polyketides
Cell Growth and Death
8.47e-5
2.64e-4
4.30e-4
2.41e-3
3.52e-3
3.70e-3
3.94e-3
4.52e-3
4.65e-3
4.97e-3
5.40e-3
6.07e-3
6.93e-3
0.013
0.016
0.017
0.019
0.021
0.035
p-value (corrected)
HIV negative
HIV positive
0.0
14.3
Mean proportion (%)
−1.5 −1.0 −0.5 0.0 0.5 1.0 1.5 2.0 2.5
Difference in mean proportions (%)

Figure S4

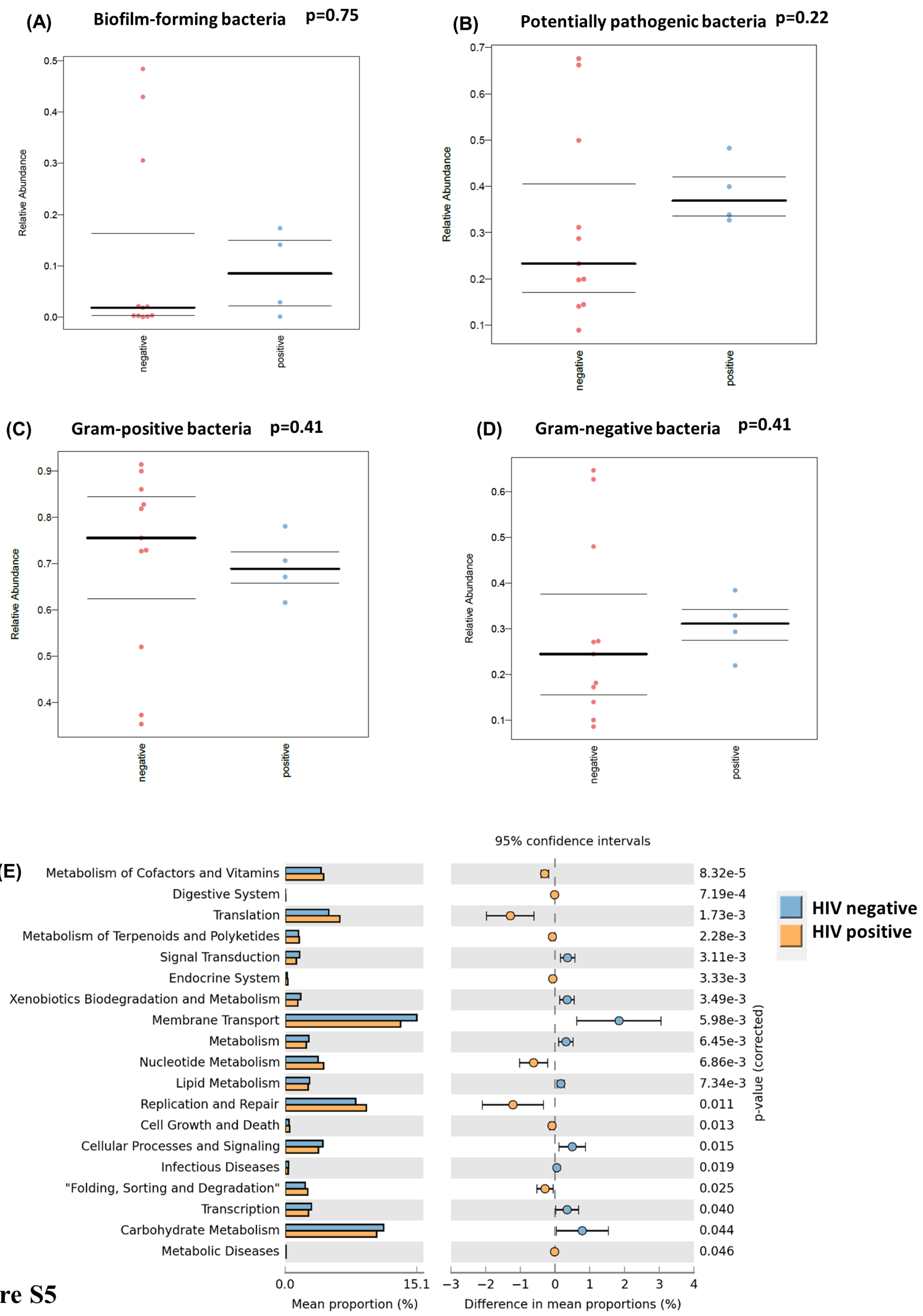
(A) Biofilm-forming bacteria p=0.75
(B) Potentially pathogenic bacteria p=0.22
(C) Gram-positive bacteria p=0.41
(D) Gram-negative bacteria p=0.41
Relative Abundance
negative
positive
(E)
95% confidence intervals
Metabolism of Cofactors and Vitamins 8.32e-5
Digestive System 7.19e-4
Translation 1.73e-3
Metabolism of Terpenoids and Polyketides 2.28e-3
Signal Transduction 3.11e-3
Endocrine System 3.33e-3
Xenobiotics Biodegradation and Metabolism 3.49e-3
Membrane Transport 5.98e-3
Metabolism 6.45e-3
Nucleotide Metabolism 6.86e-3
Lipid Metabolism 7.34e-3
Replication and Repair 0.011
Cell Growth and Death 0.013
Cellular Processes and Signaling 0.015
Infectious Diseases 0.019
"Folding, Sorting and Degradation" 0.025
Transcription 0.040
Carbohydrate Metabolism 0.044
Metabolic Diseases 0.046
Mean proportion (%)
Difference in mean proportions (%)
p-value (corrected)
HIV negative
HIV positive

Figure S5

(A)

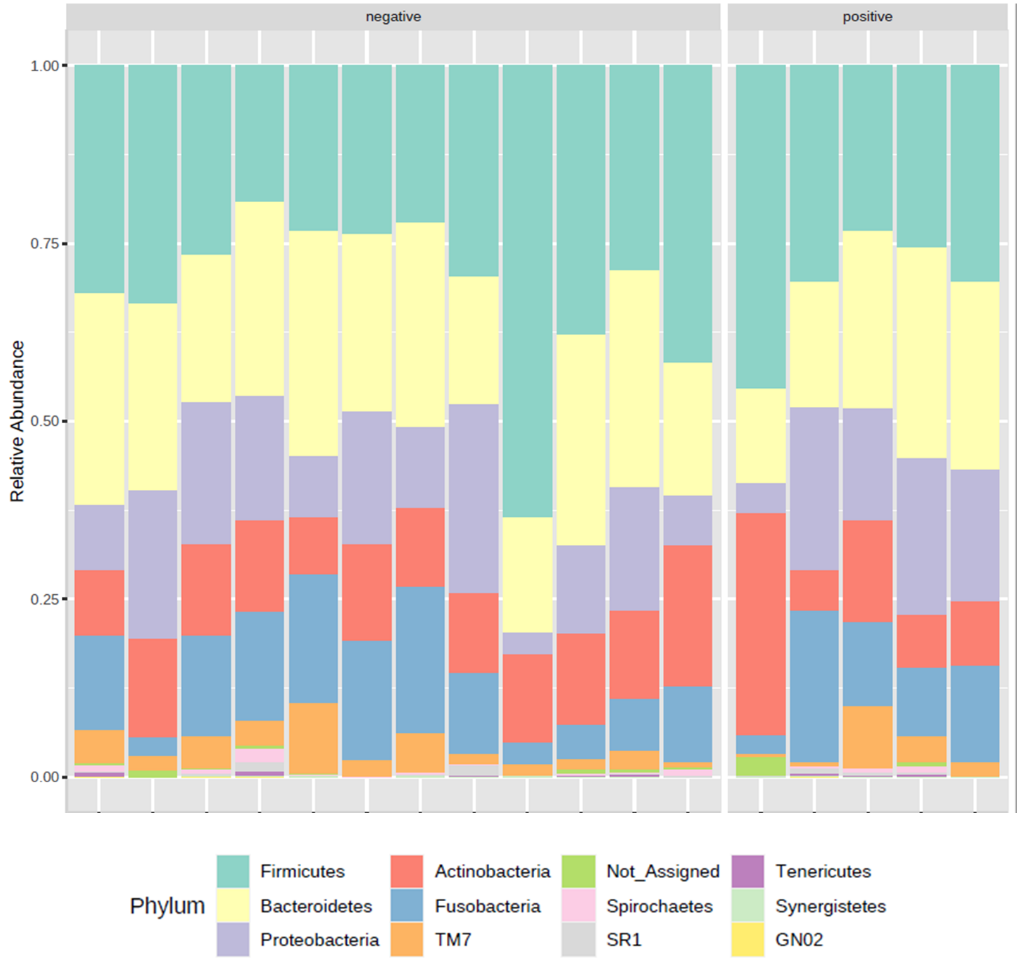
negative
positive
Relative Abundance
1.00
0.75
0.50
0.25
0.00
Phylum
Firmicutes
Bacteroidetes
Proteobacteria
Actinobacteria
Fusobacteria
TM7
Not_Assigned
Spirochaetes
SR1
Tenericutes
Synergistetes
GN02

(B)

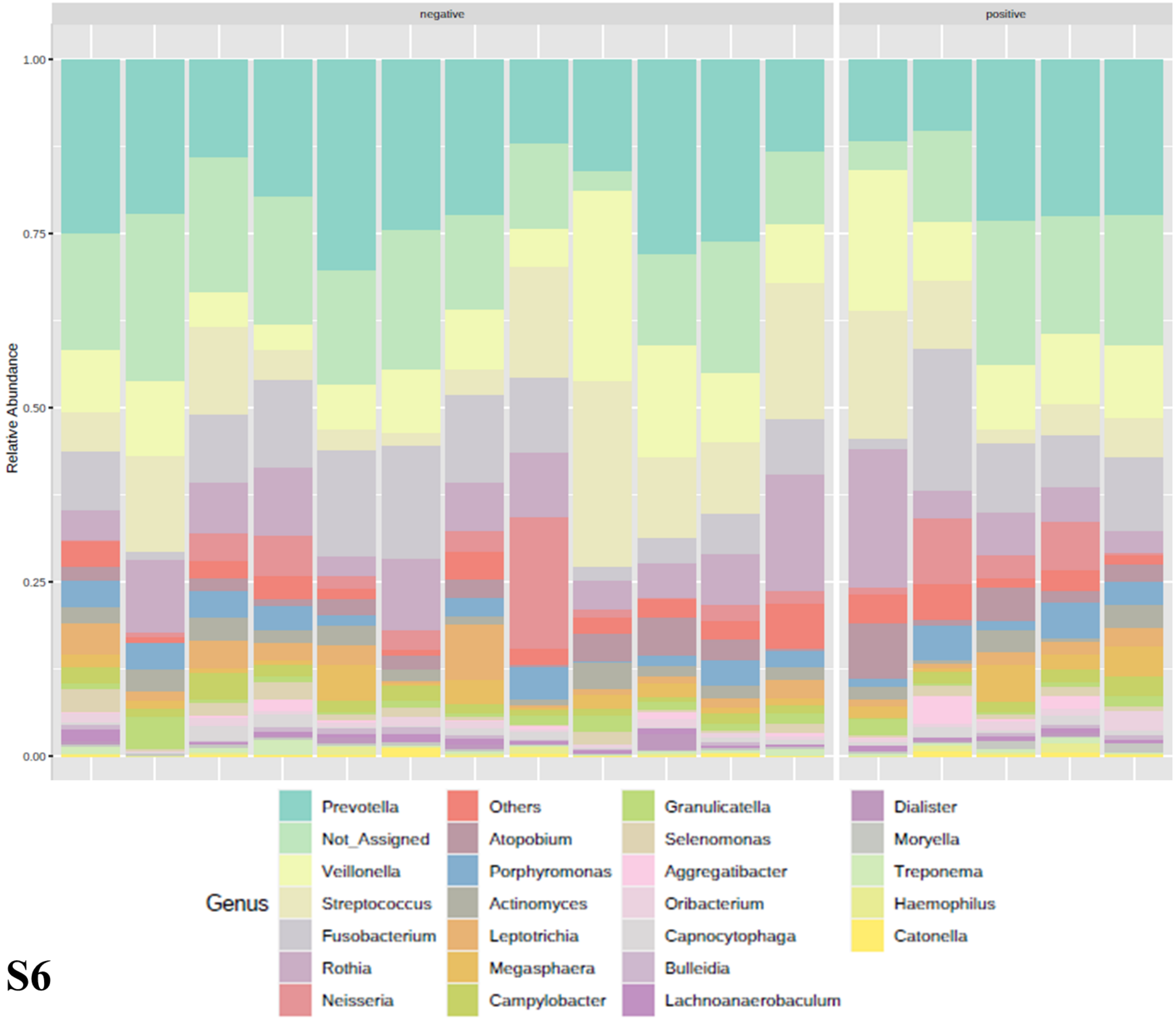
negative
positive
Relative Abundance
1.00
0.75
0.50
0.25
0.00
Genus
Prevotella
Not_Assigned
Veillonella
Streptococcus
Fusobacterium
Rothia
Neisseria
Others
Atopobium
Porphyromonas
Actinomyces
Leptotrichia
Megasphaera
Campylobacter
Granulicatella
Selenomonas
Aggregatibacter
Oribacterium
Capnocytophaga
Bulleidia
Lachnoanaerobaculum
Dialister
Moryella
Treponema
Haemophilus
Catonella

Figure S6

(A)

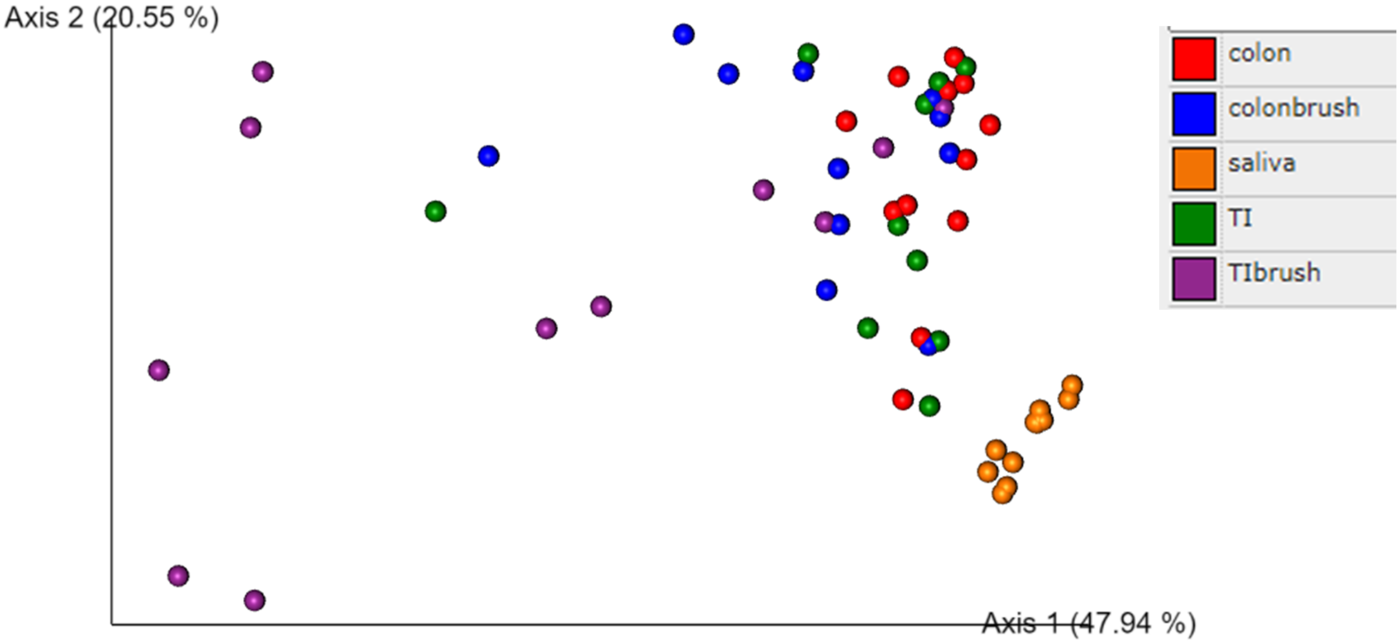
Axis 2 (20.55 %)
Axis 1 (47.94 %)
colon
colonbrush
saliva
TI
TIbrush

(B)

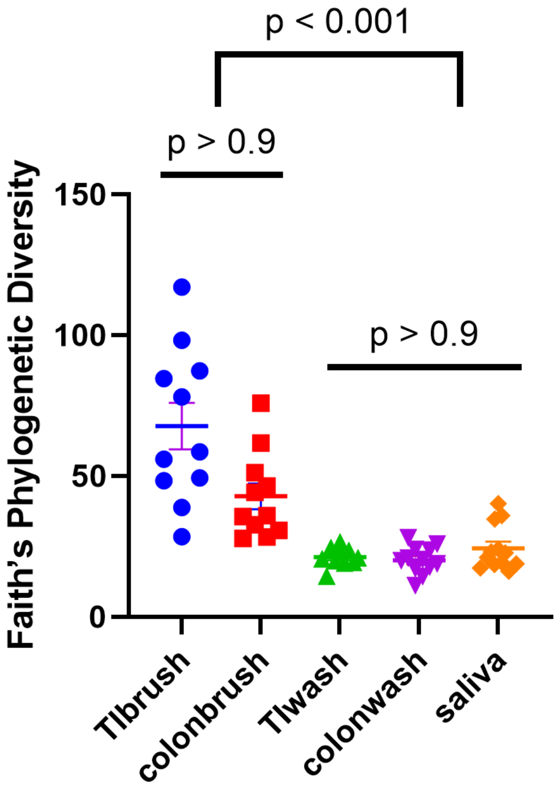
p < 0.001
p > 0.9
p > 0.9
Faith's Phylogenetic Diversity
150
100
50
0
TIbrush
colonbrush
TIwash
colonwash
saliva

Figure S7

(A)

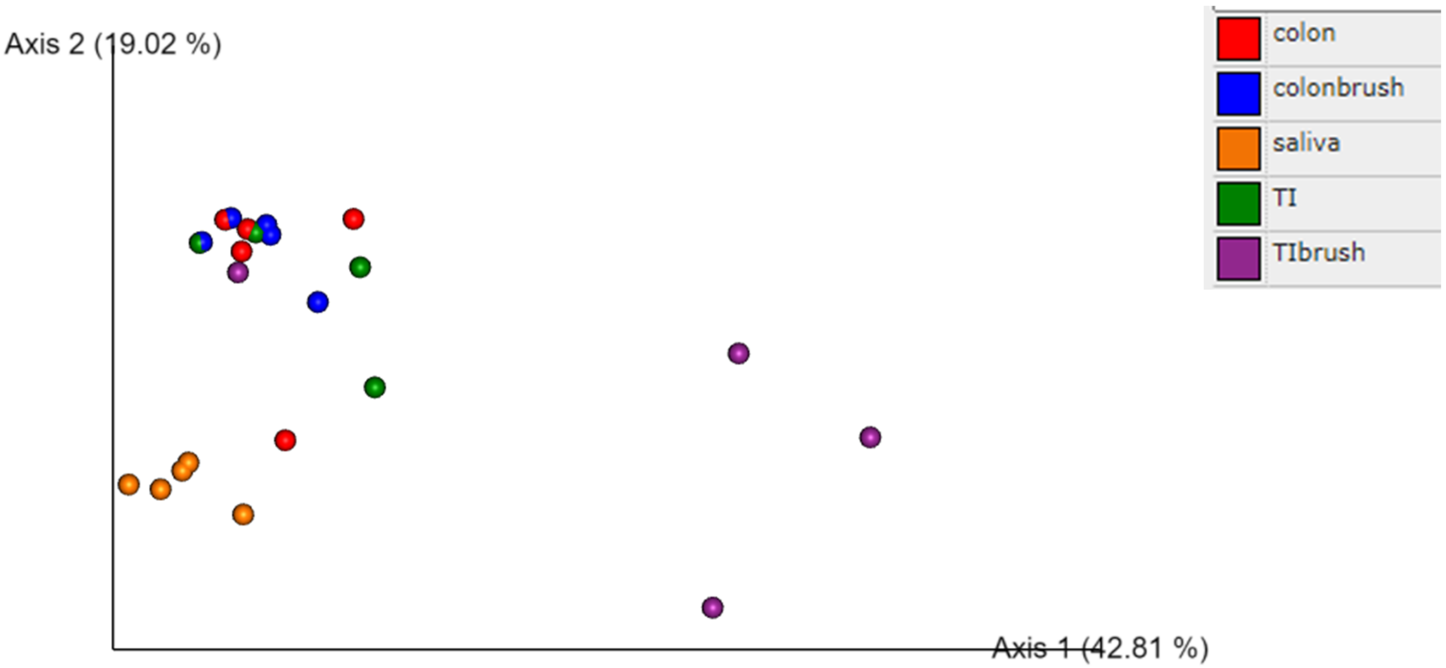
Axis 2 (19.02 %)
Axis 1 (42.81 %)
colon
colonbrush
saliva
TI
TIbrush

(B)

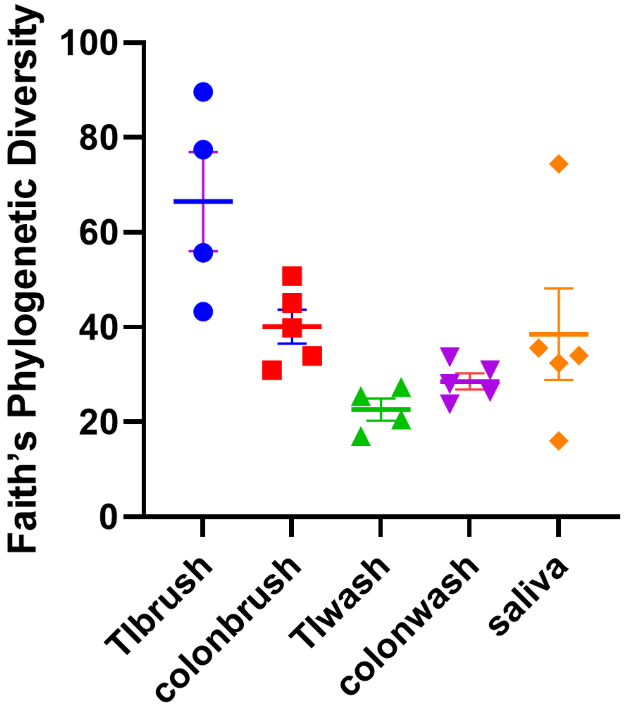
Faith's Phylogenetic Diversity
100
80
60
40
20
0
TIbrush
colonbrush
TIwash
colonwash
saliva

Figure S8

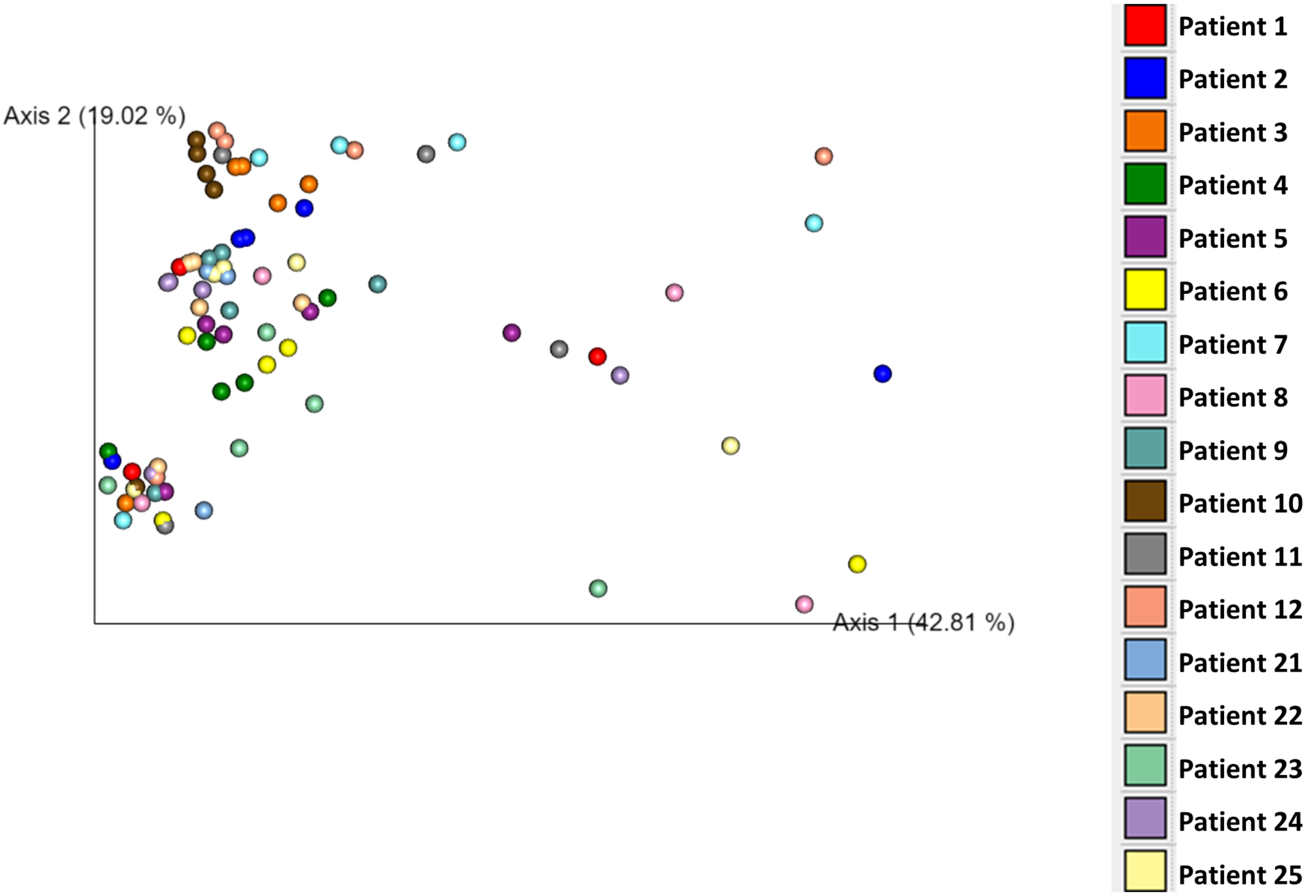

Axis 2 (19.02 %)
Axis 1 (42.81 %)
Patient 1
Patient 2
Patient 3
Patient 4
Patient 5
Patient 6
Patient 7
Patient 8
Patient 9
Patient 10
Patient 11
Patient 12
Patient 21
Patient 22
Patient 23
Patient 24
Patient 25

Figure S9
